# Stealth Fluorescence Labeling for Live Microscopy Imaging of mRNA Delivery

**DOI:** 10.1021/jacs.1c00014

**Published:** 2021-04-02

**Authors:** Tom Baladi, Jesper R. Nilsson, Audrey Gallud, Emanuele Celauro, Cécile Gasse, Fabienne Levi-Acobas, Ivo Sarac, Marcel R. Hollenstein, Anders Dahlén, Elin K. Esbjörner, L. Marcus Wilhelmsson

**Affiliations:** † Department of Chemistry and Chemical Engineering, Chemistry and Biochemistry, Chalmers University of Technology, SE-41296 Gothenburg, Sweden; ‡ Medicinal Chemistry, Research and Early Development, Cardiovascular, Renal and Metabolism, BioPharmaceuticals R&D, AstraZeneca, Gothenburg, Sweden; § Department of Biology and Biological Engineering, Chemical Biology, Chalmers University of Technology, SE-41296 Gothenburg, Sweden; ∥ Génomique Métabolique, Genoscope, Institut François Jacob, CEA, CNRS, Univ Evry, Université Paris-Saclay, 91057 Evry, France; ⊥ Department of Structural Biology and Chemistry, Laboratory for Bioorganic Chemistry of Nucleic Acids, CNRS UMR3523, Institut Pasteur, 28, Rue du Docteur Roux, 75724 Paris CEDEX 15, France

## Abstract

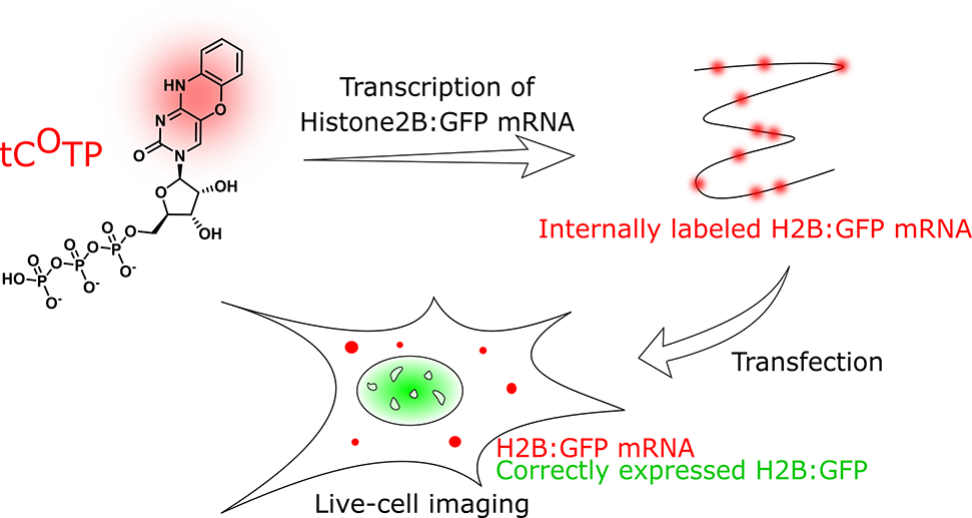

Methods for tracking RNA inside living cells without perturbing their natural interactions and functions are critical within biology and, in particular, to facilitate studies of therapeutic RNA delivery. We present a stealth labeling approach that can efficiently, and with high fidelity, generate RNA transcripts, through enzymatic incorporation of the triphosphate of tC^O^, a fluorescent tricyclic cytosine analogue. We demonstrate this by incorporation of tC^O^ in up to 100% of the natural cytosine positions of a 1.2 kb mRNA encoding for the histone H2B fused to GFP (H2B:GFP). Spectroscopic characterization of this mRNA shows that the incorporation rate of tC^O^ is similar to cytosine, which allows for efficient labeling and controlled tuning of labeling ratios for different applications. Using live cell confocal microscopy and flow cytometry, we show that the tC^O^-labeled mRNA is efficiently translated into H2B:GFP inside human cells. Hence, we not only develop the use of fluorescent base analogue labeling of nucleic acids in live-cell microscopy but also, importantly, show that the resulting transcript is translated into the correct protein. Moreover, the spectral properties of our transcripts and their translation product allow for their straightforward, simultaneous visualization in live cells. Finally, we find that chemically transfected tC^O^-labeled RNA, unlike a state-of-the-art fluorescently labeled RNA, gives rise to expression of a similar amount of protein as its natural counterpart, hence representing a methodology for studying natural, unperturbed processing of mRNA used in RNA therapeutics and in vaccines, like the ones developed against SARS-CoV-2.

## Introduction

RNA is a key molecule of life and a main active player of the central dogma of molecular biology. RNA is also a crucial regulator of gene expression via for instance micro (mi)- and small interfering (si)-RNA and through its intrinsic catalytic activity; RNA therefore plays a fundamental role in biology. It has, for these reasons, emerged as a highly promising and versatile drug modality with potential to modify cellular function at the translational level, opening up entirely new avenues to address previously undruggable targets.^1^ Increased molecular and mechanistic knowledge of biological processes that involve RNA is therefore important and requires, in many instances, new methodological tools. In the context of RNA therapeutics, cellular delivery remains a major challenge, and better understanding of cellular uptake, endosomal release, and cytosolic delivery of RNAs is needed to unleash their full potential.^2−4^ A major problem in this regard relates to the challenge of directly visualizing RNA molecules as they are taken up, processed, and subsequently released into the cytosol.^5^

Recent advances in RNA imaging have generated a broad spectrum of tools and probes by which RNA can be analyzed and quantified, but they generally rely on the use of heavily modified externally labeled oligonucleotides with chemical and physical properties that are significantly different from their natural counterparts. Cyanine dyes such as Cy3 and Cy5 conjugated, for instance, via strain-promoted cycloaddition linkers remain the most common labeling choice,^6,7^ even though their bulkiness and hydrophobicity significantly impede both transcription and translation^8^ (has also been noted by TriLink Biotechnologies, one of the main manufacturers of externally labeled mRNA) of mRNA. Development of more universally applicable RNA labeling schemes and probes that are minimally perturbing to RNA’s functions and compatible with live-cell fluorescence imaging is therefore needed and may become as crucial for the RNA field as the discovery and understanding of the green fluorescent protein (GFP) has been for proteins.^9−11^

A major challenge in RNA imaging is to develop new methods and probes that are functional in living cells.^12^ While methods reliant on cell fixation such as fluorescence in situ hybridization (FISH) can visualize and quantify endogenous mRNA interactions with astounding specificity and single-molecule resolution,^13^ they fall short with respect to capturing the spatiotemporal and conformational dynamics that are important to RNA function. The development of FRET-pair-functionalized antisense oligonucleotides (ASOs)^14,15^ is highly interesting in this regard, but requires binding of at least two ASOs to the mRNA target, which may sterically block protein-binding sites, hinder mRNA translation, or induce tertiary structure formation; in practice the method has also mainly been used on fixed cells. To visualize RNA in live cells, probes are often conjugated to a vehicle, such as a DNA nanocages^16^ or gold nanoparticles,^17^ before delivery. Another emerging strategy is to fuse the RNA of interest to an aptamer sequence that binds fluorescent dyes in situ,^18^ but this can also adversely influence translation efficiency and RNA–protein interactions.^19^ To reduce the risks of interfering with enzymatic processes, chimeric mRNAs have been developed with multiple stem-loop structures downstream of the STOP codon, which serve as binding sites for fluorescent fusion proteins.^20−22^ While versatile, all these labeling strategies impact profoundly on the physicochemical properties and molecular weights of RNAs and therefore risk significantly perturbing their natural function, spatiotemporal distribution, and transport.

Additional less perturbing strategies for labeling mRNA have therefore been developed, relying for example on click chemistry approaches with azido-functionalized 5′-cap analogues,^23^ 3′-polyA tails,^24^ or nucleotides^25^ enabling in-cell post-transcriptional labeling of mRNA. Similarly, Ziemniak et al. have produced a range of fluorescent 5′-cap analogues that are compatible with both transcription and translation.^26^ However, the resulting mRNA products only carry one single label and have therefore not been possible to visualize inside cells, limiting the applicability of this method.

Fluorescent nucleobase analogues (FBAs) have emerged as attractive labels for DNA and RNA. However, even though we and others have significantly improved FBAs with respect to brightness, excitation, and emission to facilitate their use in fluorescence microscopy,^27−29^ significant challenges have remained regarding development of FBAs that are sufficiently enzyme-compatible to be effectively processed during transcription and translation.^30^ FBAs have the advantage of being internal fluorophores, with relatively small chemical modifications to the natural base that they replace. Furthermore, their design enables normal base-pairing and -stacking of the target nucleic acid. FBAs are therefore considered to be native-like fluorescent labels and have been extensively used in vitro to probe nucleic acid structure and behavior. We have, for example, designed FBA interbase FRET pairs to obtain detailed information on the structure and base orientation in DNA^31^ and RNA,^32,33^ and others have used FBAs to study biophysically ribosome-mediated codon:anticodon base-pair formations.^34^ A handful of studies indicate that FBAs can be incorporated into RNA via cell-free transcription, resulting in for example ca. 800 nucleotide (nt) RNA strands with a modified cystosine^35^ or short transcripts with fluorescent isomorphic guanine^36,37^ and uridine.^38,39^ However, none of these studies have proven that FBA transcripts can be translated, and FBA-labeled RNAs have not yet been used in biological applications or to visualize RNA molecules inside living cells. This progress is needed to translate FBAs from useful in vitro probes to functional tools for chemical and medical biology.^30^

**Figure 1:**
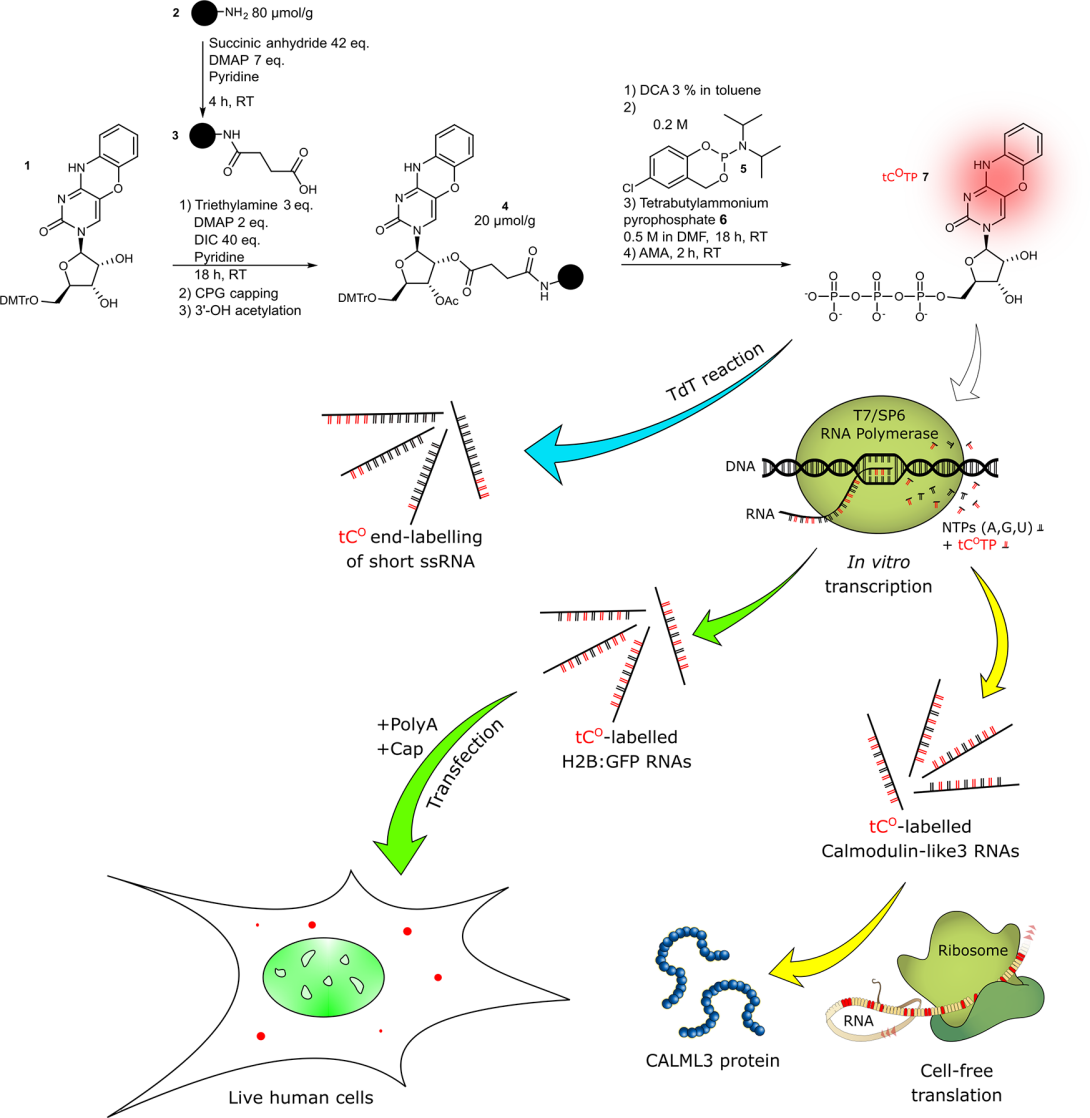
Schematic outline of the study detailing the synthesis of the main building block of the study, the fluorescent tricyclic cytosine analogue tC^O^ triphosphate (**7**) and its use. Blue pathway: End-labeling of ssRNA sequences using a terminal deoxynucleotidyl transferase (TdT). Green pathway: Cell-free transcription into H2B:GFP mRNA, transfection into human cells, and live-cell imaging of both the GFP (translation product) and tC^O^ (mRNA). Yellow pathway: Cell-free transcription into calmodulin-like3 RNA followed by cell-free translation into the CALML3 protein. Color code: tC^O^ in red, GFP in green, native nucleosides in black or beige.

In this study, we demonstrate that the fluorescent tricyclic cytosine analogue 1,3-diaza-2-oxophenoxazine (tC^O^; Abs_max_ = 369 nm; Em_max_ = 457 nm; ε_369_ = 9370 M^–1^ cm^–1^; ⟨Φ_F_⟩ = 0.24)^32^ can be enzymatically incorporated in high numbers into RNA via end-labeling reactions as well as cell-free transcription. We furthermore show that it is possible to exchange all natural cytosines in a 1.2 kb long mRNA for tC^O^ (Figure 1) and retain translation competence both in vitro and in human cells. We also demonstrate, for the first time, that an FBA-labeled mRNA can be sufficiently fluorescent to be directly visualized by confocal microscopy in a living human cell and used to study mRNA delivery and protein translation in a drug delivery context.

This presents a significant advance to the FBA and RNA imaging fields and a new powerful tool to enable effective visualization of RNA and thereby enable studies of RNA function, trafficking, and localization in a variety of cellular contexts, including for example drug delivery, virus processing, and exosome biology.

## Results and Discussion

### Synthesis of the Tricyclic Cytosine Analogue Triphosphate

The tC^O^ ribonucleoside triphosphate has, unlike the corresponding nucleoside, never been synthesized, and, hence, we needed to establish a synthetic route toward our target molecule. Since the Yoshikawa^40^ and Ludwig–Eckstein^41^ conditions were published in 1969 and 1989, respectively, a plethora of methods have been proposed for the triphosphorylation of nucleosides,^42−45^ but no generic method exists to effectively synthesize and purify ribonucleoside triphosphates in the high yields that are required for practical use in biochemical applications.

To overcome this “hit-and-miss” aspect of triphosphate synthesis, we developed a new synthesis scheme that requires no preliminary protection of the 2′- and 3′-positions and that facilitates purification of the final product (Figure 1). In our hands, this new method allowed the synthesis of the tC^O^ ribonucleoside triphosphate used herein and two additional nucleobase-modified ribonucleoside triphosphates (manuscripts in preparation) in equally good yields, and we envision it could therefore become a generic and convenient route toward any modified nucleoside triphosphate.

The tC^O^ ribonucleoside triphosphate was synthesized from a solid-supported ribonucleoside, a strategy that has never been reported for modified nucleobases, but attempted with some success for unmodified nucleobases with 2′-OMe backbone protection. Our synthetic scheme relies on phosphoramidite chemistry, which involves the use of *cyclo*Sal-phosphoramidite **5** and bis(tetrabutylammonium) dihydrogen pyrophosphate **6** (Figure 1). This approach was developed by Meier et al.^46^ to achieve efficient 5′-triphosphorylation of short solid support-bound DNA and RNA oligonucleotides in moderate to good yields. In our hands, both Krupp’s^47^ and Meier’s^46^ solid-phase triphosphorylation methods yielded the product, although Meier’s gave a higher yield. To the best of our knowledge, the *cyclo*Sal-phosphoramidite method on a solid support described herein has never been applied to a single ribonucleoside before, let alone to produce a modified nucleoside triphosphate.

Briefly, a long-chain alkylamino controlled-porosity glass (CPG) support was functionalized with a succinyl moiety. Protected ribonucleoside **1** was synthesized according to a method by Füchtbauer et al.^32^ and attached to the succinylated support via ester bond formation (Figure 1). The resulting ribonucleoside **4** on a solid support could be stored in the dark at room temperature, with no degradation observed over three months. Interestingly, intermediate **4** could also be synthesized via succinylation of ribonucleoside **1** in solution followed by coupling with the amino support **2**. In both cases, the starting nucleoside could be used unprotected at the 2′- and 3′-positions, thus eluding the need for additional protection steps, which makes our method straightforward. Subsequently, triphosphorylation of ribonucleoside **4** was performed using the *cyclo*Sal method. After triphosphorylation, support-bound triphosphate was deacetylated and cleaved from the CPG support using ammonium hydroxide/methylamine (AMA) for 2 h at room temperature. Subsequent reverse phase or ion-exchange chromatography allowed the desired triphosphate **7** in a triphosphorylation yield of 60% and with a high UV purity of 99%. Importantly, up to 85% of the unreacted nucleoside **1** could conveniently be recovered by precipitation from the first reaction crude, compensating for the low loading achieved. Considering this, our triphosphorylation method gives an overall yield of up to 30% of the tC^O^ ribonucleoside triphosphate, which is higher than most solution-based alternatives.

### Cell-Free Enzymatic Incorporation of tC^O^ into RNA

The tC^O^ ribonucleoside triphosphate (tC^O^TP) was used to produce fluorescently labeled RNA via two different enzymatic methods.

**Figure 2:**
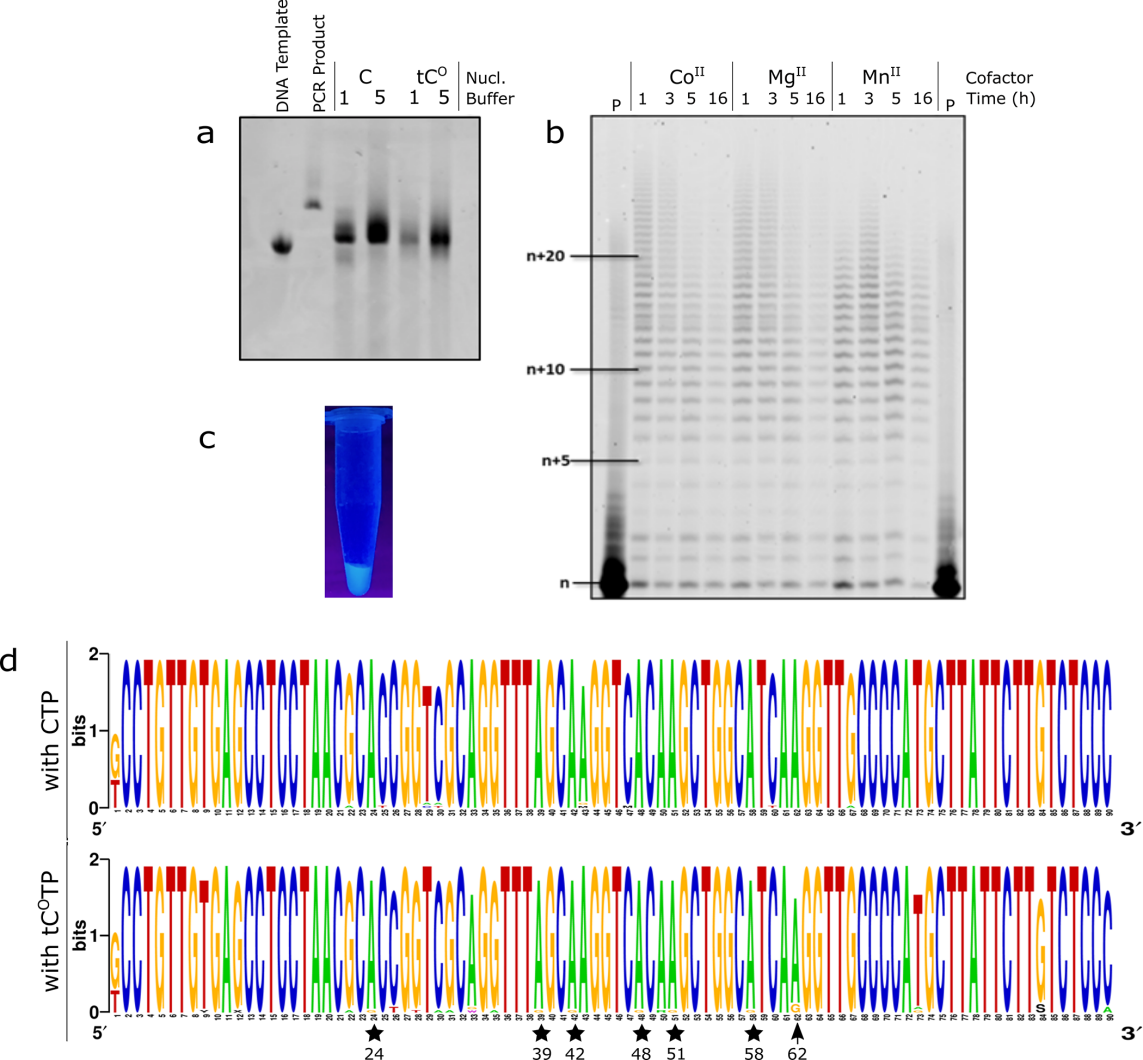
Enzymatic incorporation of the modified triphosphate by cell-free transcription with short templates, sequencing of reverse transcriptase products, and TdT-mediated labeling. (a) Gel image (PAGE 10%; visualization by Midori green) of RNA products from 6 h of T7 RNA polymerase-assisted transcription using the D1 Library as a DNA template. See Methods for buffer composition. (b) Gel image (PAGE 20%; visualization by phosphorimager) of the products from TdT-mediated end-labeling of oligonucleotide TdT1 using tC^O^TP. The lowest band labeled “n” represents the unreacted TdT1 oligonucleotide, and all above bands are different products with increasing additions of tC^O^ to the tail. P: control reaction in absence of polymerase. (c) Picture of UV-light-irradiated solution of purified TdT3 RNA end-labeled with tC^O^. (d) Sequence logo from the cloning–sequencing protocol of reverse transcription products from the modified T12.3 RNA (top: products from a 6 h transcription reaction with CTP; bottom: products from a 6 h transcription reaction with tC^O^TP). Lower (stars) and higher (arrow) frequency A to G transversions are highlighted.

First, we tail-labeled short RNA oligonucleotides using the terminal deoxynucleotidyl transferase enzyme (TdT, Figure 1, blue arrow), which catalyzes template-independent addition of random nucleotides to 3′-overhangs in both DNA and RNA.^48,49^ Starting from a 17-mer ssRNA (TdT1, see Supplementary Table 1 for sequence) we demonstrate successful addition of multiple adjacent tC^O^s (from one to >25) on nearly all RNA primers (Figure 2b). The addition was equally effective with Co(II), Mg(II), or Mn(II) as cofactor in accord with the normally reported function of TdT,^48^ supporting a native-like enzymatic processing of tC^O^. TdT-mediated tail-labeling of longer (50 nt, TdT2, Supplementary Table 1) ssRNA sequences was also successful, and the main products were more uniform, containing typically one or two tC^O^ (Supplementary Figure 1). The lower processivity found for this longer RNA could be an effect of its length or of its 3′-sequence but more likely due to the fact that the TdT is a DNA polymerase that prefers deoxyribonucleotides both as substrates and as templates.^48^ The fluorescence originating from a tC^O^ tail-labeled RNA was clearly observable by the naked eye upon UV irradiation (Figure 2c). This proves that it is possible to use tC^O^ to site-specifically end-label RNA of various length, which represents an advantage in terms of enabling dual labeling of RNA strands, where combinations of tC^O^ with other base- or backbone-modified fluorescent markers will enable monitoring of, for example, in vivo stability.

We also tested the capacity of T7 RNA polymerase to incorporate tC^O^TP into short (20 nt/90 nt; Supplementary Table 1) RNA transcripts, observing successful transcription without premature transcription termination even upon complete replacement of canonical CTP with tC^O^TP (Figure 2a, Supplementary Figure 2a, and Supplementary Figure 3). This confirms that tC^O^TP is readily accepted also by the T7 polymerase as a substrate. To further assess the enzymatic processing of tC^O^TP, we tested its propensity to be misinserted opposite a single deoxyadenosine downstream of the T7 promotor sequence of a guanosine-free DNA template (Supplementary Table 1; DNA3, DNA4). Transcription reactions were run in the presence of either CTP or tC^O^TP and in the absence of UTP (Supplementary Figure 2b). With the first template (DNA3), no noticeable difference was observed in the reactivity of tC^O^TP and CTP, whereas a mismatch closer to the T7 promoter sequence (DNA4) resulted in slightly fewer aborted transcripts and hence more full-length transcripts with tC^O^TP compared to native CTP. This suggests a minor (20% based on densiometric analysis, see Supporting Information) increase in error frequency with tC^O^TP. A previous report by Stengel et al. used a structurally related tricyclic cytosine analogue, tC, which carries a bulkier sulfur atom instead of an oxygen at position 5 of the pyrimidone ring, to produce RNA transcripts. Their reaction proceeded with a considerable formation of mismatched tC–A base pairs (a discrimination factor of 40 against was reported).^35^ As a further comparison to this, we tested the fidelity of incorporation of tC^O^TP by reverse transcription (Supplementary Figure 3). After PCR amplification and A-tailing reaction, the resulting amplicons were ligated to the pGEM T vector and transfected into beta 2033 competent *E. coli* cells, and plasmids stemming from white colonies were subjected to Illumina sequencing. Multiple alignment analysis revealed a mere 2-fold higher frequency of misincorporation with tC^O^TP compared to CTP (28 vs 16 point mutations in respectively 34 and 38 analyzed sequences; Supplementary Figures 4 and 5); significant A to G transversions were observed at position 62 (3 mutations, Figure 2d and Supplementary Figure 6) and a lower number of transversions at positions 24, 39, 42, 48, 51, 58 (1 mutation). In combination with the above-mentioned minor difference in abortion frequency (20% or lower) found between CTP and tC^O^TP, this suggests an overall lower level of misincorporation for tC^O^TP than for the analogue reported by Stengel et al. Altogether, this shows that the fluorescent tC^O^TP nucleotide is well-tolerated by both TdT and T7 RNA polymerases, with the latter only marginally increasing the incorporation error rate in up to 50 nt long RNAs. In this sense, tC^O^TP behaves better than the majority of previously modified nucleotides,^35,36,50−52^ including bulkier triphosphate analogues.^53,54^ It has been reported that the T7 RNA polymerase binds the incoming nucleotide substrate in an open conformation,^55^ which could enable it to accommodate our modified base.

### Cell-Free Enzymatic Incorporation of tC^O^ into Full-Length mRNA Transcript

**Figure 3:**
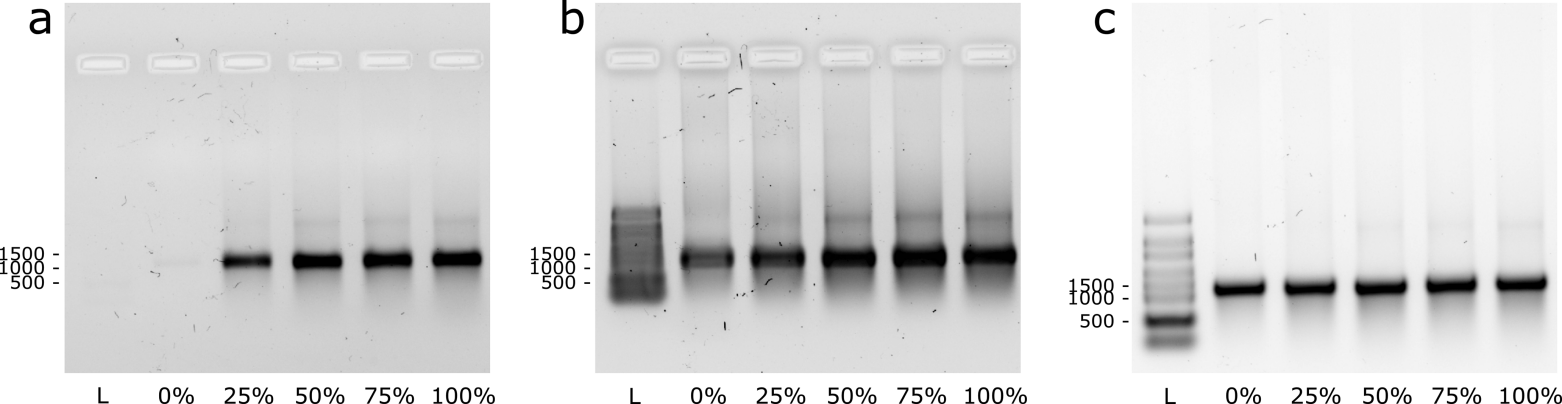
Incorporation of tC^O^ into full-length mRNA by T7 RNA polymerase assisted cell-free transcription. Denaturing agarose bleach gels showing RNA transcripts formed at five different tC^O^TP/CTP ratios (0–100%). Direct visualization of tC^O^ fluorescence (a) and after ethidium bromide staining (b). RNA samples were heat-denatured (65 °C for 5 min, 1.5% bleach in the gel) prior to loading. (c) Same RNA transcripts upon harsher denaturation (70 °C for 10 min, 2% bleach in the gel). The RiboRuler High Range RNA ladder was used.

We next extended the in vitro transcription to test if it was possible to produce full-length, translationally active mRNAs with different degrees of tC^O^. Transcription of long RNA containing a fluorescent base analogue has only been reported once,^35^ but their ca. 800 nt transcript was not active in reverse transcription and no translation activity was reported, suggesting that the transcripts were nonfunctional. Moreover, they showed that on increasing the amounts of their fluorescent tricyclic cytosine nucleoside triphosphate, the transcription reaction led to shorter transcripts, suggesting premature termination. Apart from being a fluorophore with superior brightness we envisioned that tC^O^, with its oxygen in the middle ring instead of the considerably bulkier sulfur (vdW radius of sulfur is 20–30% larger, resulting in an approximately 100% larger occupied volume) for the tricyclic cytosine used by Stengel et al., would represent a better cytosine analogue that would minimize perturbations to biological processes. We used a DNA template encoding for histone protein H2B fused to GFP (H2B:GFP) to produce mRNA via cell-free transcription. The template was codon optimized (Supplementary Figure 7) to limit the number of C repeats to limit self-quenching of adjacent tC^O^ moieties^56^ (vide infra). This optimization could also have the additional positive effect that consecutive incorporations of tC^O^s would be limited; previous work indeed suggested difficulties for DNA polymerases to incorporate consecutive dtC^O^TPs.^57^ However, we also observed in vitro transcription of a nonoptimized calmodulin mRNA (vide infra) even at 100% tC^O^TP (0% CTP) despite this transcript containing three CCCC repeats. We observed efficient transcription and tC^O^ incorporation with both T7 and SP6 RNA polymerases (Figure 1, first green arrow) at tC^O^TP/canonical CTP ratios ranging from 0 to 100% (full replacement), as demonstrated by agarose bleach gel electrophoresis of the resulting transcripts (Figure 3a, Supplementary Figure 8a). The RNA transcripts appeared as one single band, with a migration corresponding to the expected 1247 nt mRNA product (H2B:GFP), demonstrating that full-length mRNA was formed. The tC^O^-containing mRNA bands could be directly visualized upon 302 nm excitation using a conventional gel scanner (Figure 3a); the increasing band intensities with increasing tC^O^TP/CTP reaction ratio supported successful concentration-dependent incorporation of tC^O^. Ethidium bromide staining (Figure 3a, right) revealed similar amounts of RNA in all reactions, suggesting that tC^O^ incorporation does not reduce the cell-free transcription reaction yield. Furthermore, unlike the RNA product formation reported by Stengel et al.,^35^ no shorter transcripts were observed, strengthening the conclusion that the T7 RNA polymerase processes tC^O^TP correctly and without premature abortion. Faint higher order bands were apparent with all tC^O^-containing RNA transcripts (Figure 3b and Supplementary Figure 8b) but could be effectively removed by heat denaturation (Figure 3c). The intensity of the higher order band appears to increase slightly with tC^O^ content, which could reflect the increased hydrophobicity introduced by this modified base. Importantly, we demonstrate that tC^O^ can be successfully incorporated into full-length mRNA and that this reaction proceeds effectively even when canonical CTP is completely replaced with tC^O^TP, suggesting that this base analogue is highly mimetic of its natural counterpart.

### Incorporation Efficiency and Spectroscopic Properties of tC^O^-Containing mRNA

**Figure 4:**
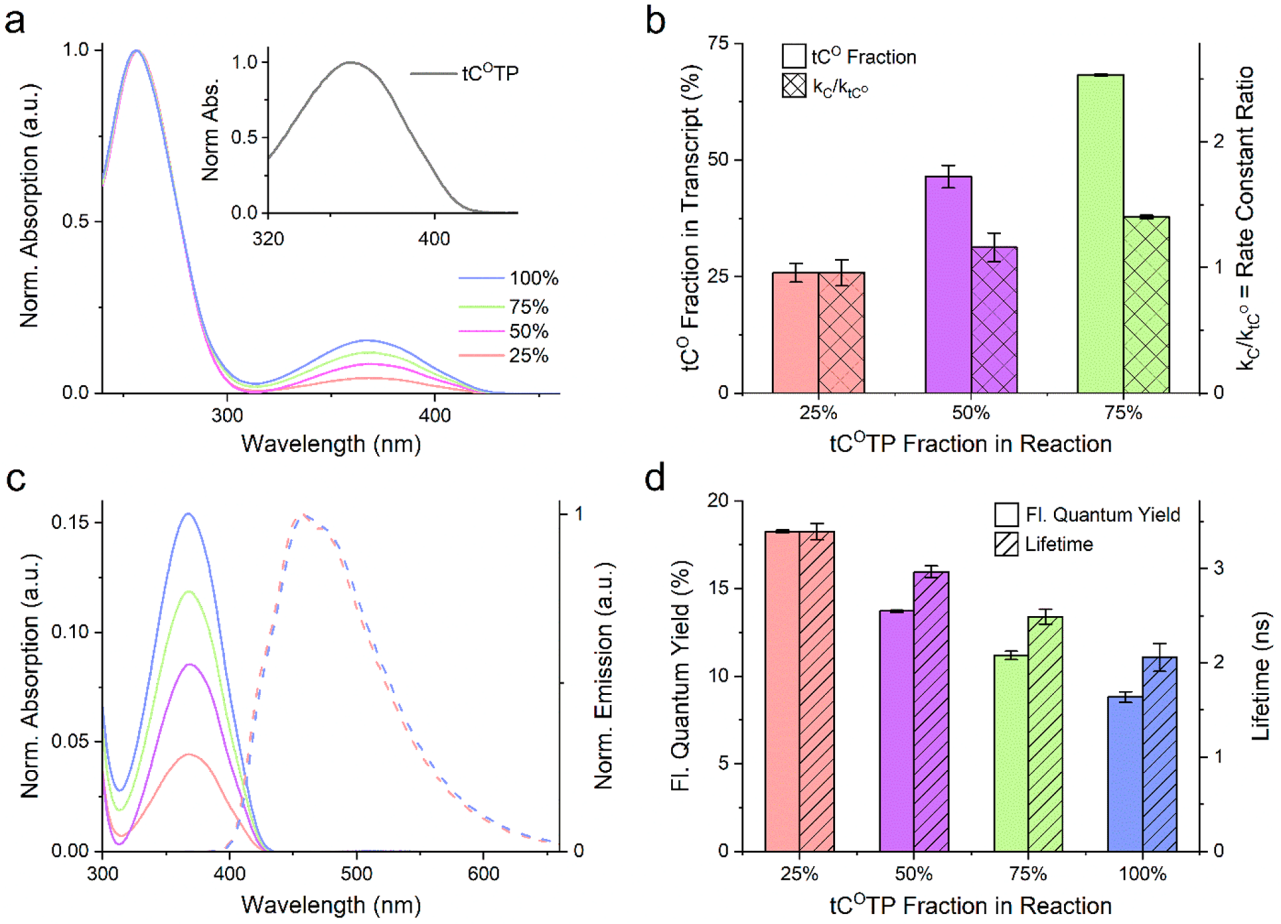
Spectroscopic characterization of cell-free synthesized tC^O^-modified RNA transcripts. Four reactions charged with different molar fractions of tC^O^TP (blue: 100%, green: 75%, magenta: 50%, and red: 25%) in the total cytosine triphosphate pool (tC^O^TP + CTP) were performed. The product transcripts were purified to wash out unreacted triphosphates prior to characterization. All reactions were performed as independent duplicates, and the results are presented as mean ± standard deviation. (a) UV–vis absorption spectra normalized to *A* = 1 at the RNA band, ca. 260 nm. Inset: tC^O^TP absorption normalized to *A* = 1 at the tC^O^ band λ_max_ (360 nm). (b) Plain bars: Fraction of incorporated tC^O^ (relative to the total amount of incorporated cytosines, i.e., tC^O^ + C) in the transcripts. Checkered bars: Ratio of first-order reaction rate constants for CTP vs tC^O^TP consumption. (c) Solid lines: UV–vis absorption spectra (normalized to *A* = 1 at the RNA band, ca. 260 nm) showing the tC^O^ band centered at 368–369 nm. Dashed lines: Emission spectra normalized to *I* = 1 at λ_max_ (457 and 459 nm for the 25% and 100% transcript, respectively). For clarity, the emission spectra for the 50% and 75% reactions were omitted. (d) Plain bars: Fluorescence quantum yields. Striped bars: Fluorescence lifetime.

We used absorption and fluorescence spectroscopy to quantify the incorporation efficiency of tC^O^TP compared to canonical CTP, following purification of the RNA transcripts using a commercial cleanup kit to remove unreacted tC^O^TP. Absorption spectra (Figure 4a) of tC^O^-containing transcripts show an absorption peak at 370 nm, consistent with its reported spectrum inside RNA.^32^ The relative magnitude of this peak compared to the RNA absorption at 260 nm (reflecting total concentration) confirms that the degree of tC^O^ incorporation is concentration-dependent and allowed us to determine the relative rate constants for the incorporation of CTP and tC^O^TP (*k*_C_ and *k*_tCO_, respectively, see Methods for details). The ratios between the rate constants *k*_C_/*k*_tCO_ were close to or slightly above unity for all transcripts (0.96–1.4, Figure 4b), suggesting strongly that T7 RNA polymerase discriminates only marginally between CTP and tC^O^TP. Our observation of equal incorporation efficiencies for tC^O^TP and CTP differs from other published studies, where the corresponding incorporation ratio was found to be lower than 0.6 (and down to 0.13) for d(tC^O^TP) incorporation into DNA and analogues d(tCTP) into DNA and tCTP into RNA.^35,57,58^ This suggests that tC^O^ is a good nature-mimic with minimal perturbing effects on the transcriptional process. Furthermore, a trivial but noteworthy consequence of the determined rate constants is that the tC^O^ incorporation degree in our mRNA matches extremely well the percentage of tC^O^TP added to the transcription reaction (Figure 4b).

In quantitative fluorescence-based cell analyses, it is important that fluorescence intensity is proportional to probe concentration. We therefore examined the emissive behavior of tC^O^ in the mRNA transcripts as a function of its degree of incorporation. We report a minor red-shift of the emission spectrum (ca. 4 nm) with increasing tC^O^ incorporation (Figure 4c), whereas more significant effects were observed on fluorescence quantum yields and lifetimes (Figure 4d). The figure shows that the quantum yield drops from 0.18 at 25% incorporation to 0.09 at 100% incorporation, consistent with a corresponding drop in fluorescence lifetime from 4.3 ns to 3.2 ns. We ascribe this to electronic coupling of molecular states of the tC^O^ fluorophore and a self-quenching effect^56^ due to increased adjacency between tC^O^s^57^ (vide supra and Supplementary Figure 9). The self-quenching at high tC^O^ incorporation affects the relative brightness of the transcripts, despite codon optimization, such that maximum emission is achieved at 75%, although with relatively modest improvement compared to 50% substitution (Supplementary Figure 9). However, as shown below, a substitution level of 25% was sufficient to visualize lipid-complexed tC^O^-labeled mRNA (Figure 6c), indicating what we believe to be a suitable labeling range for most cell applications. This is also comparable to the 25% substitution level of U-positions with a widely used commercial Cy5-mRNA delivered by TriLink, vide infra. Importantly, due to the minor differences in incorporation preference between tC^O^TP and CTP, we show that it is possible to tune, with accuracy, the labeling density of mRNA to optimize the relationship between brightness and biological function (for example in translation as discussed below).

**Figure 5:**
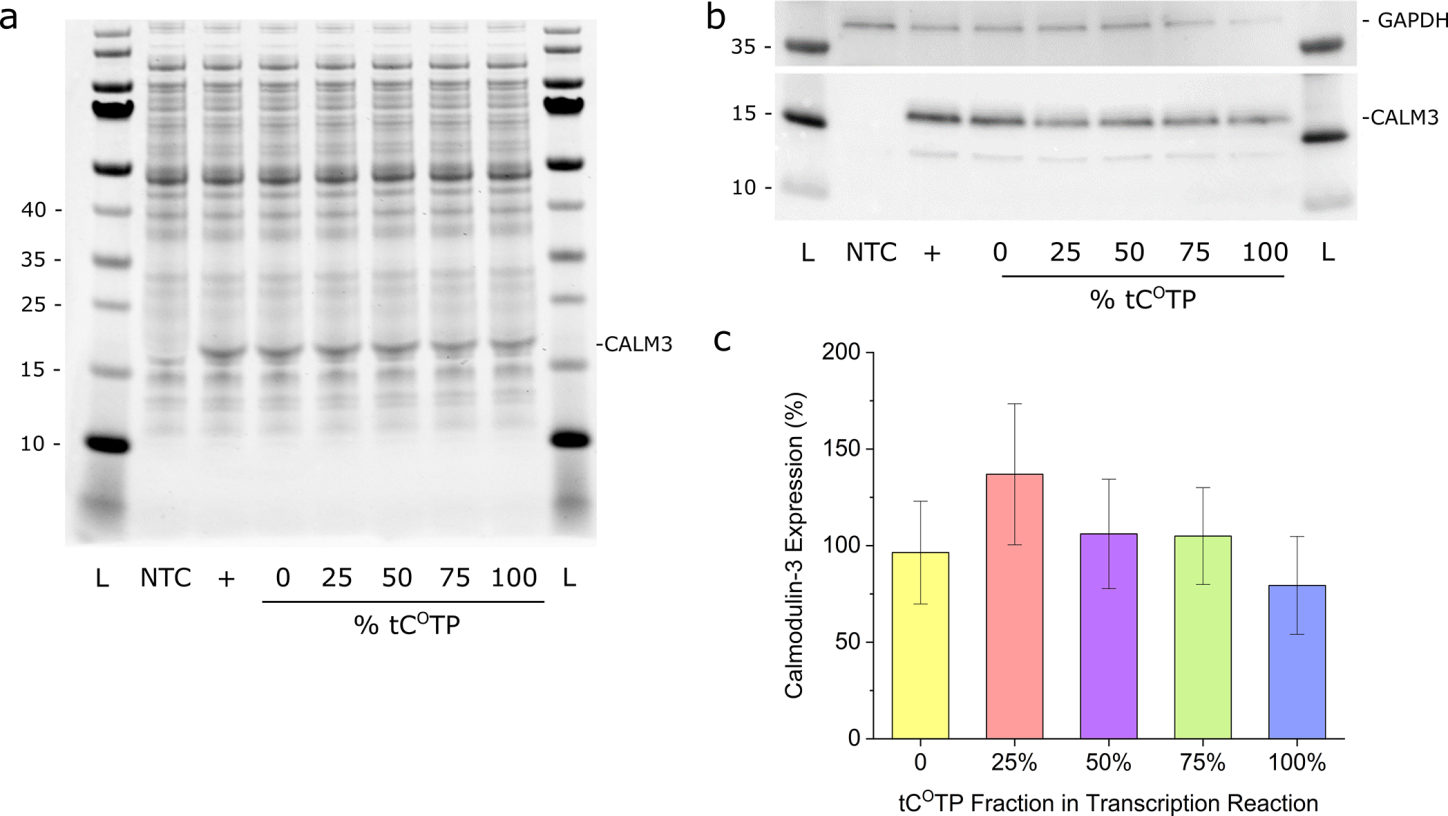
Cell-free translation of calmodulin-3 (CALM3) mRNA. (a) Coomassie staining and (b) Western blot (WB) of the in vitro translation reactions. NTC: no template control; + : kit template RNA control. The PageRuler Prestained Protein Ladder was used. (c) Quantification by WB and densitometry analysis (mean % of control ± standard deviation; *n* = 3). One-way ANOVA with means comparison using Tukey’s post hoc test showed no significant effect of tC^O^ content in the mRNA transcripts on protein translation (*p* < 0.05).

**Figure 6:**
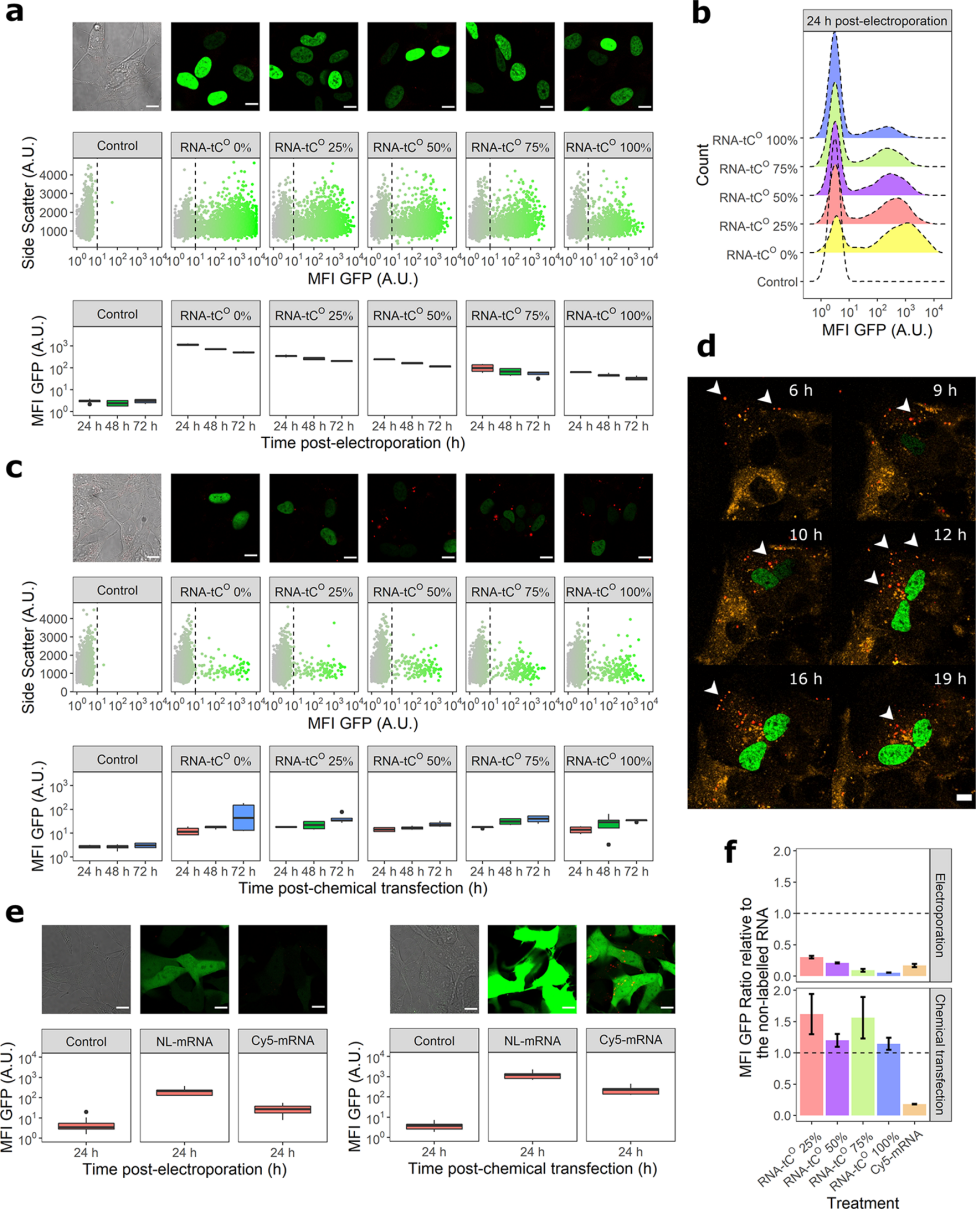
Translation and visualization of tC^O^-labeled mRNA in human SH-SY5Y cells. The mRNA translation was monitored based on the fluorescence intensity of the encoded H2B:GFP protein using confocal microscopy and flow cytometry. (a) mRNA translation following electroporation; (top) confocal images (3× enlargements, scale bars: 10 μm) recorded 24 h postelectroporation, (middle) flow cytometry scatter plots 24 h postelectroporation, and (bottom) mean cellular GFP fluorescence intensity (MFI GFP ± standard deviation) of all counted cells at 24, 48, and 72 h postelectroporation. (b) Representative MFI GFP histograms corresponding to the distributions in (a). (c) mRNA translation following chemical transfection (lipofection), corresponding to the data shown in (a). (Top) Confocal images (3× enlargements, scale bars: 10 μm) recorded 48 h postchemical transfection, (middle) flow cytometry scatter plots 48 h postelectroporation, and (bottom) MFI GFP of all counted cells at 24, 48, and 72 h postchemical transfection. (d) Snap-shot images from a confocal time-lapse experiment to monitor the intracellular trafficking of 75% tC^O^-labeled mRNA (red) introduced, by chemical transfection, into cells with an overexpression of mRFP-Rab5 to label early endosomes (orange). Resulting expression of H2B-GFP protein in the nucleus is shown in green. White arrows indicate discrete mRNA–lipid complexes; scale bars: 10 μm. Supplementary Movie 1 shows the full time lapse. (e) mRNA translation of cyanine5-labeled (Cy5) eGFP encoding mRNAs (TriLink) 24 h post-transfection (electroporation or chemical transfection). NL; nonlabeled. Scale bars: 10 μm. (f) Impact of tC^O^ or Cy5 incorporation on mRNA translation, represented as the ratio of cellular MFI GFP of the labeled mRNA relative to the cellular MFI GFP for the corresponding nonlabeled RNA. All cell experiments were performed in three biological replicates.

### In Vitro Translation of tC^O^-Labeled mRNA

We next demonstrated that tC^O^-labeled mRNA is functional in translation, providing the first evidence that this is possible using an FBA-modified mRNA. We used a commercial transcription–translation kit to produce calmodulin-3 under cell-free conditions in bacterial lysates (Figure 1, yellow arrows). mRNA, with the same tC^O^TP/CTP ratios as used above (0 to 100% of tC^O^TP), was produced using the calmodulin-3 DNA template plasmid provided with the kit, i.e., without any codon optimization. The expression of calmodulin-3 protein was first confirmed by SDS PAGE followed by Coomassie staining (Figure 5a); the calmodulin-3 band intensities for all samples with tC^O^-modified mRNA are within 10% of that of the 0% tC^O^ control, suggesting that the FBA has no significant effect on translation. The calmodulin-3 production was further verified and quantified by Western blot (Figure 5b). Densiometric analysis of the calmodulin-3 bands (normalized to the intensities of the GAPDH loading controls) of three replicate samples showed that the protein yields from tC^O^-labeled mRNAs were within 80–137% of the unlabeled control (Figure 5c); one-way Anova analysis with Tukey’s post hoc test showed that the observed variations in sample means were not statistically significant; *p* < 0.05. Still, judging from the trends in the Coomassie-stained gel and WB data it may be possible that the translation of the 100% tC^O^-labeled mRNA could be slightly impaired. Altogether, however, the in vitro translation data suggest that tC^O^, unlike other sugar-^52^ or base-modified^59^ nucleotide analogues, behaves very similar to canonical cytosine with respect to ribosomal processing and translation in cell-free systems. This shows that tC^O^ therefore functions as a novel fluorescent mimic of cytosine in both transcription and translation.

### Translation and Visualization of tC^O^-Labeled mRNA in Human Cells

We used two different approaches (electroporation and chemical transfection more closely mimicking drug delivery) to introduce tC^O^-labeled mRNAs into human cells and test their in-cell translatability. The transcripts used were 5′-capped and 3′-protected by poly adenylation (by ca. 300 nt) to avoid degradation; we found this to be necessary also for tC^O^-containing mRNA, suggesting that the FBA does not alter its stability (see further below).

We observed, by confocal fluorescence microscopy of live SH-SY5Y cells, the expression of a correctly localized (nuclear) and folded (fluorescent) protein product following both electroporation and chemical transfection of the tC^O^-labeled mRNAs (Figure 6a and c, Supplementary Figure 10a and b), suggesting functional processing of the FBA-modified mRNAs by human ribosomal machineries as well as low frequency of misincorporations of tC^O^ into RNA during cell-free transcription as reported in Figure 2.

We thereafter used flow cytometry to quantify the translation of protein, based on mean cellular GFP fluorescence intensities (Figure 6, Supplementary Figure 10). Electroporation was used to introduce mRNA directly to the cytosol and resulted in significantly higher transfection efficiency (i.e., number of GFP-positive cells) than chemical transfection with Lipofectamine (Supplementary Figure 10c). We observed that the transfection efficiency following electroporation decreased with increasing tC^O^ content of the mRNA, an effect that rendered an effective lowering of the mean fluorescence intensity of the cell populations electroporated with mRNA containing tC^O^. Although this could indicate that tC^O^ impedes in-cell translation, our results using chemical transfection (see below) suggest this is rather an effect of how the mRNA was introduced. It is possible that the increased hydrophobicity of tC^O^-labeled mRNAs in combination with the high invasiveness and electric fields of electroporation caused some tC^O^ mRNA degradation and/or imposed secondary structures that prevented translation. A slightly higher propensity to adopt secondary structures in vitro is indicated from the gel analysis in Figure 3. In any case, the effect was not associated with toxicity (Supplementary Figure 10g) and the fluorescence signal persisted over 72 h postelectroporation, although during this time the GFP fluorescence gradually decreased (Figure 6a). This is likely a combined effect of cell division (the doubling time of SH-SY5Y cells under our experimental conditions is approximately 24 h) and cytosolic mRNA degradation. Importantly, none of these effects appear affected by tC^O^ content, suggesting that the tC^O^-labeled mRNA that reaches the cytosol in a functional form is processed as the corresponding nonlabeled mRNA.

Chemical transfection using Lipofectamine resulted in considerably fewer GFP-positive cells (Supplementary Figure 10c), but, importantly, no tC^O^-dependent effects on either transfection efficiency or mean cellular GFP fluorescence were observed (Figure 6c). This is in agreement with the cell-free translation experiments (Figure 5) and suggests that tC^0^-labeled mRNA, if appropriately delivered, can yield as much cellular protein product as the corresponding nonlabeled mRNA (see Figure 6f and further below). Following chemical transfection, the mean fluorescence intensity increases over time (Figure 6c), reflecting a continuous Lipofectamine-mediated and tC^O^-independent endocytic uptake and escape of mRNA cargo to the cytosol.

The complexation of the tC^O^-labeled mRNA with Lipofectamine enabled its direct visualization inside cells using live cell confocal microscopy (Figure 6c, red puncta). This represents the first observation of FBA-labeled nucleic acids inside a living cell and demonstrates that tC^O^ is bright enough to be tracked following 405 nm laser excitation and yielded sufficient contrast over the autofluorescent cellular background that is typical to live cells. We therefore proceeded to visualize, in real time, both the uptake and subsequent translation of an FBA-modified mRNA by time-lapse recordings of cells overexpressing an early endosome biomarker (mRFP-Rab5, orange) (Figure 6d). We observed temporal colocalization (Supplementary Movie 1) of the tC^O^ signal with mRFP-Rab5 proteins, highlighting the fact that the mRNA transits through the early endosome following endocytosis. Importantly, our results show a successful new methodology that enables not only simultaneous spatiotemporal tracking of the uptake and trafficking of mRNA during cellular delivery but also the appearance of its correctly localized and folded translation product.

Finally, we compared the translation of the tC^O^-labeled H2B-GFP encoding mRNA to that of the nonlabeled variant (Figure 6f) to illustrate the effect of the FBA modification on in-cell translation (which is apparent with electroporation but not with chemical transfection as also discussed above). We, thereafter, did the same experiment with a commercial eGFP-encoding Cy5-labeled mRNA (with ca. 25% of all U positions carrying the Cy5 dye) relating its translation to that of the corresponding nonlabeled eGFP control (Figure 6e). Despite the low labeling density, the Cy5-labeled mRNA has only ca. 20% of the translation capacity of its corresponding eGFP-encoding nonlabeled mRNA following both electroporation and chemical transfection (Figure 6f). This is consistent with previous in vitro translation studies that confirm the translation-impeding nature of the Cy5 modification.^8^ Although the tC^O^- and Cy5-labeled mRNAs encode for different proteins, the normalization against their respective nonlabeled controls may still give an indication of the relative effects on translation of the two label types. Notable, in this respect, is that Lipofectamine-delivered tC^O^-labeled mRNAs appear to function similarly to their controls, whereas the Cy5-labeled variant is significantly translation impeded. This could indicate an improved translatability of mRNA carrying our fluorescent FBA.

## Conclusion

In this study we demonstrate that tC^O^, a fluorescent cytosine base analogue with moderate chemical modification, in a remarkable way takes the role of natural cytosine in a number of biochemical processes. This FBA is correctly recognized by several enzymatic machineries, including RNA polymerases, reverse transcriptase, and bacterial as well as human ribosomes. We have also developed a robust, generic, and affordable synthesis method to produce the triphosphate variants of unnatural nucleotides, presenting the first successful synthesis of the tC^O^TP nucleoside triphosphate. This chemical development is critical to enable the use of tC^O^ and other FBAs in biochemical and biological applications.

We demonstrate that tC^O^ can be incorporated into short and long sequences of RNA via both end-labeling and cell-free transcription, which enables versatile introduction of FBAs in both flanking and functional segments of different kinds of RNA. Importantly, we have managed to incorporate tC^O^ in up to 100% of natural cytosine positions of a full-length 1.2 kb mRNA, without any premature abortions. Analysis of the transcription products demonstrated that tC^O^ is incorporated into RNA virtually as efficiently as native CTP and therefore constitutes a true nature-mimicking fluorescent modification in this respect.

Astoundingly, we also found that tC^O^-labeled mRNA is translated into its correctly folded and localized protein product, both in vitro and in live cells. This has not been shown for FBA-modified mRNA before; in fact previous published work using the cytosine analogue tC failed to generate translation-competent mRNA even starting from a considerably shorter (0.8 kB) template. In addition, both in vitro translation experiments and chemical transfection of cells indicate that tC^O^-labeled mRNAs can translate equally well as the corresponding nonlabeled control, suggesting that this type of internal label can indeed be less disrupting to mRNA function compared to a Cy5-label. This points to the flexibility and versatility of the herein discovered tC^O^-labeling method, which offers opportunity to tune labeling density to optimize brightness and distribution of fluorophores along the mRNA sequence. Moreover, when using Cy5-labeled mRNA to study delivery, the modus in the field is to mix in a fraction of the labeled mRNA with the corresponding unlabeled mRNA, a procedure that increases the risk of incorrectly reported rates and levels of delivery and translation even further, something that now can be avoided with our nature-mimicking labeling technique.

Finally, we present the first example of an FBA-labeled nucleic acid that can be directly visualized in live cells, showing that tC^O^’s brightness and absorption at 405 nm are sufficient to overcome previous limitations with FBA probes in biological applications.^30^ Moreover, we demonstrate how this conveniently allows for spatiotemporal monitoring of uptake, trafficking, and organelle colocalization of chemically transfected mRNA in a live cell model with simultaneous detection of its translation into H2B:GFP protein.

We envision that our straightforward approach for introducing nonperturbing fluorescent labels into RNA will be an excellent addition to existing imaging tools, applicable for elucidating trafficking mechanisms such as endosomal escape and exosome formation, both of which are of fundamental importance for pharmaceutical development. Applying both TdT end-labeling and T7 transcription strategies also holds significant combined potential, as it would enable selective and site-specific incorporation of dual labels to allow for, for example, FRET applications. We believe that the development reported here will benefit pharmaceutical industry, clinical laboratories, and academic partners aiming at furthering their understanding of uptake and endosomal escape mechanisms and allow them to take vital steps toward new and improved delivery strategies for next-generation nucleic acid-based drugs as well as further development of the recently successful mRNA-based vaccines.

## Supporting Information Available

Supporting Movie 1 shows the internalization of the 75% tC^O^-labeled mRNA and its expression over 55 h in Huh7 cells (1 s = 1.1 h) (AVI)
Characterization of tC^O^TP, all experimental procedures, sequence alignments, additional electrophoresis gels, confocal images, and flow cytometry data (PDF)

## Supplementary Material

ja1c00014_si_001.avi
