# Supplementary material for: Stealth Fluorescence Labeling for Live Microscopy Imaging of mRNA Delivery
Source: J Am Chem Soc. 2021 Apr 2;143(14):5413–24. doi: 10.1021/jacs.1c00014 (PMC8154517; doi:10.1021/jacs.1c00014)
Supplement: Supplementary file 2 — ja1c00014_si_002.pdf [file ja1c00014_si_002.pdf]

SUPPORTING INFORMATION

Stealth fluorescence labeling for live microscopy imaging of mRNA delivery

Tom Baladi[a,b], Jesper R. Nilsson[b], Audrey Gallud[c], Emanuele Celauro[c], Cécile Gasse[d], Fabienne Levi-Acobas[e], Ivo Sarac[e], Marcel R. Hollenstein[e], Anders Dahlén[b], Elin K. Esbjörner[c] & L. Marcus Wilhelmsson[a]

a Medicinal Chemistry, Research and Early Development, Cardiovascular, Renal and Metabolism, BioPharmaceuticals R&D, AstraZeneca, Gothenburg, Sweden
b Department of Chemistry and Chemical Engineering, Chemistry and Biochemistry, Chalmers University of Technology, SE-41296, Gothenburg, Sweden
c Department of Biology and Biological Engineering, Chemical Biology, Chalmers University of Technology, SE-41296, Gothenburg, Sweden
d Institute of Systems & Synthetic Biology, Xenome Team, 5 rue Henri Desbruères Genopole Campus 1, University of Evry, F-91030 Evry, France
e Institut Pasteur, Department of Structural Biology and Chemistry, Laboratory for Bioorganic Chemistry of Nucleic Acids, CNRS UMR3523, 28, rue du Docteur Roux, 75724 Paris CEDEX 15, France

Experimental procedures

Chemistry S2

Spectroscopy S10

Biochemistry S14

Supplementary Table 1. S25

Supplementary Figure 1. S26

Supplementary Figure 2. S27

Supplementary Figure 3. S28

Supplementary Figure 4. S29

Supplementary Figure 5. S30

Supplementary Figure 6. S31

Supplementary Figure 7. S32

Supplementary Figure 8. S33

Supplementary Figure 9. S34

Supplementary Figure 10. S35

Supplementary Table 2. S36

Experimental procedures: Chemistry

Commercially available reagents were used without further purification. The following reagents used for the triphosphorylation were bought from Sigma-Aldrich: DCA deblock for ÄKTA, CAP A for ÄKTA, CAP B1 and B2 for ÄKTA, BTT Activator. ^{1}H (500 MHz) and ^{13}C (126 MHz) NMR spectra were recorded at 300 K on a Bruker 500 MHz system equipped with a CryoProbe. ^{31}P (202 MHz) NMR spectra were recorded at 300 K on a Bruker 500 MHz system. All shifts are recorded in ppm relative to the deuterated solvent (DMSO-d_6, $CDCl_3$ or D_2O).

3-((2*R*,3*R*,4*S*,5*R*)-5-((bis(4-methoxyphenyl)(phenyl)methoxy)methyl)-3,4-dihydroxytetrahydrofuran-2-yl)-3*H*-benzo[*b*]pyrimido[4,5-*e*][1,4]oxazin-2(10*H*)-one 1: Compound was prepared according to the literature.[1] MS (ESI$^-$) $[M-H]^-$=634.5. ^{1}H NMR (500 MHz, DMSO-d_6) δ 10.61 (bs, 1H), 7.42 (d, *J* = 7.7 Hz, 2H), 7.27 – 7.35 (m, 7H), 7.22 (t, *J* = 7.1 Hz, 1H), 6.90 (dd, *J* = 8.6, 4.2 Hz, 4H), 6.75 – 6.87 (m, 3H), 6.46 (d, *J* = 7.8 Hz, 1H), 5.71 (d, *J* = 3.6 Hz, 1H), 5.49 (bs, 1H), 5.18 (bs, 1H), 4.08 (d, *J* = 5.3 Hz, 1H), 4.04 (s, 1H), 3.94 (s, 1H), 3.71 (s, 3H), 3.70 (s, 3H), 3.29 (d, *J* = 4.8 Hz, 1H), 3.16 (d, *J* = 9.1 Hz, 1H). Spectroscopic data in agreement with the literature.

Preparation of CPG solid support 3: The support **2** (1 g, 0.08 mmol) was activated by shaking in trichloroacetic acid 3% in DCE (8 mL, 0.08 mmol) for 18 h. The activated support was then filtered off and washed with 9:1 triethylamine:diisopropylethylamine (20 mL), dichloromethane (20 mL) and diethyl ether (20 mL). The activated support was dried under vacuum for 2 days before use. Subsequently, the support (1 g, 0.08 mmol), succinic anhydride (0.345 g, 3.44 mmol) and *N*,*N*-dimethylpyridin-4-amine (0.070 g, 0.57 mmol) were suspended in dry Pyridine (3 mL) under N2. The reaction mixture was then gently shaken at RT for 4 h. After 4 h, solvent was filtered off and the support washed successively with pyridine (20 mL), dichloromethane (20 mL), diethyl ether (20 mL) and air-dried. Negative ninhydrin test on a small portion of support proved full succinylation. Succinylated CPG could thereafter be kept at room temperature for several months.

CPG-supported (2*R*,3*R*,4*R*,5*R*)-5-((bis(4-methoxyphenyl)(phenyl)methoxy)methyl)-4-hydroxy-2-(2-oxo-2,10-dihydro-3*H*-benzo[*b*]pyrimido[4,5-*e*][1,4]oxazin-3-yl)tetrahydrofuran-3-yl acetate 4: In a 10 mL syringe with PTFE filter, succinylated support **3** (1.420 g, 82 µmol/g, 0.12 mmol), DMAP (0.028 g, 0.23 mmol), DIC (719 µl, 4.64 mmol), **1** (0.076 g, 0.12 mmol) and triethylamine (49 µl, 0.35 mmol) were suspended pyridine (5 mL). The mixture was gently shaken for 18 h at RT. After 18 h, the syringe was purged and the support washed with pyridine (5 mL), dichloromethane (5 mL) and diethyl ether. Subsequently, in the same syringe, DMAP (0.028 g, 0.23 mmol), DIC (719 µl, 4.64 mmol), triethylamine (49 µl, 0.35 mmol) and 2,3,4,5,6-pentachlorophenol (0.309 g, 1.16 mmol) were added to the support and suspended in pyridine (4 mL). The mixture was gently shaken for 4 h at RT before a solution of piperidine (2 mL, 20% in DMF – for capping of the unreacted carboxylic acids on the support) was added for 1 min (longer exposure time will reduce loading as piperidine cleaves the ester bonds with the nucleoside), then quickly washed away with DMF (3x5 mL), dichloromethane (5 mL) and diethyl ether (5 mL). Finally, the resin was shaken in a CAP A + CAP B mix (50/50 v/v) for 2 hours under argon atmosphere, then washed with DMF (5 mL), dichloromethane (5 mL), diethyl ether (5 mL) and argon-dried (final loading: 13 µmol/g – determined by reading optical density of a DMT solution cleaved from a weighed amount of support - ε=70000 $M^{-1}.cm^{-1}$ at 498 nm). Final loading can be increased by performing a second coupling with **1** in the same conditions before capping (typical loading after second coupling 15-20 µmol/g). Concentrating the reaction mixture and washing the residue multiple times with water and diethyl ether allows recovery of nearly 85 % of unreacted nucleoside **1**.

6-chloro-*N*,*N*-diisopropyl-4*H*-benzo[*d*][1,3,2]dioxaphosphinin-2-amine 5: Compound was prepared according to the literature. [2] Briefly, 5-chlorosalicylic acid was reduced with LAH (0.5 equiv.) at -20 °C and the resulting 5-chlorosalicylic alcohol was cyclized into 2,6-dichloro-4*H*-benzo[*d*][1,3,2]dioxaphosphinine using PCl_3 (1.2 equiv.) and triethylamine (2.3 equiv.) at -20 °C under argon. Low temperature and use of triethylamine as the base were decisive in avoiding rapid and quantitative Arbuzov rearrangement of the

desired product into the more stable 2,5-dichloro-3*H*-benzo[*d*][1,2]oxaphosphole 2-oxide. The crude 2,6-dichloro-4*H*-benzo[*d*][1,3,2]dioxaphosphinine was subsquently treated with diisopropylamine (3 equiv.) for 2 h at room temperature. The mixture was then filtered under argon, concentrated to dryness and taken in 20 % diisopropylamine in heptane. Quick filtration on a small silica gel plug allowed desired compound **5** as a colorless oil, crystallizing over time at -20 °C. Any attempt of more thorough column chromatography on compound **5** would lead to quantitative Arbuzov rearrangement. ^{1}H NMR (500 MHz, DMSO-d_6) δ = 7.23 (dd, *J* = 8.6, 2.6 Hz, 1H), 7.20 (d, *J* = 2.4 Hz, 1H), 6.92 (d, *J* = 8.6 Hz, 1H), 5.06 (dd, *J* = 14.7, 5.2 Hz, 1H), 4.89 (dd, *J* = 19.6, 14.8 Hz, 1H), 3.53 – 3.63 (m, 2H), 1.15 – 1.19 (dd, J = 8.0, 7.0 Hz, 12H).^{31}P NMR (202 MHz, DMSO-d_6) δ = 136.00 (s, 1P). Spectroscopic data in agreement with the literature.[3]

bis(tetrabutylammonium) dihydrogen diphosphate 6: Compound was prepared according to the literature.[3] ^{1}H NMR (500 MHz, D_2O) δ 3.04 – 3.13 (m, 16H), 1.53 (bs, 16H), 1.24 (h, *J*=7.3, 16H), 0.83 (t, *J*=7.4, 24H). ^{31}P NMR (202 MHz, D_2O) δ = -10.78 (s, 2P). Spectroscopic data in agreement with the literature.

((2*R*,3*S*,4*R*,5*R*)-3,4-dihydroxy-5-(2-oxo-2,10-dihydro-3H-benzo[*b*]pyrimido[4,5-*e*][1,4]oxazin-3-yl)tetrahydrofuran-2-yl)methyl triphosphate 7: Reaction was performed in a 5 mL syringe with PTFE filter loaded with **4** (800 mg, 0.016 mmol) under an argon atmosphere and shaking.

Steps were performed as following:

5’-DMT removal: the support was washed with a flow of DCA deblock until the filtrate was colorless, then washed with ACN (5 x 5 mL).

Coupling: *N*,*N*-diisopropyl-4*H*-benzo[*d*][1,3,2]dioxaphosphinin-2-amine **5** (345 mg, 1.36 mmol) was dissolved in 4.8 mL ACN and reacted portionwise with the support (3 equal couplings with reaction times

60 s – 60 s – 90 s respectively). To each coupling, BTT activator (2.4 mL) was also added. The support was subsequently washed with ACN (3x5 mL).

Oxidation: Pyridine/Water/Iodine (9/1/12.7 v/v/w, 5mL) for 45 s, followed by ACN wash (3x5 mL) and drying of the support in an argon flow.

Triphosphorylation: Two injections of bis(tetrabutylammonium) dihydrogen diphosphate 6 (0.5 M, 5 ml) for 15 min and 18 hours, respectively. The support was subsequently rinsed with DMF (5 mL), water (3x5 mL), ACN (5 mL) and then dried in an argon flow.

Cleavage and Purification: Cleavage of the triphosphate was done in 2 h at room temperature with AMA (50/50 v/v mix of 23 % aq. NH_4OH and 40 % aq. methylamine, 5 mL). After 2 hours, the AMA filtrate was purged in a round-bottom flask and the support was rinsed 3 times with 23% aq. NH_4OH solution. After freeze-drying of the mixture, purification by HPLC (Waters Acquity HSS T3 column, 2.1x50 mm, 0.4 mL/min, 2 to 99 % 50 mM NH_4OAc in water 80:20 EtOH) was performed to allow compound **7** (5.6 mg, 62.0 % determined from UV absorbance) as a light-yellow solid (ammonium salt). The same level of purity could be achieved with ion-exchange HPLC using a semi-preparative Dionex DNAPac PA100 column (9 x 250 mm) on an ÄKTA pure 25 HPLC system using a gradient from water to 20 % 1M NH_4HCO_4 (pH 7.8) in 30 min at a flow rate of 4 mL/min.

HRMS (ESI-TOF) *m/z* calc. for $C_{15}H_{18}N_3O_{15}P_3$ $[M+H]^+$: 574.0029, found : 574.0013; *m/z* calc. for $C_{15}H_{18}N_3O_{15}P_3$ $[M-H]^-$:571.9878, found: 571.9872.

^{1}H NMR (500 MHz, D_2O) δ 7.44 (s, 1H), 6.84 – 6.94 (m, 3H), 6.79 (dd, *J* = 7.5, 1.7 Hz, 1H), 5.91 (d, *J* = 4.9 Hz, 1H), 4.36 (t, *J* = 4.8 Hz, 1H), 4.29 (t, *J* = 5.1 Hz, 1H), 4.25 (d, *J* = 4.1 Hz, 3H).

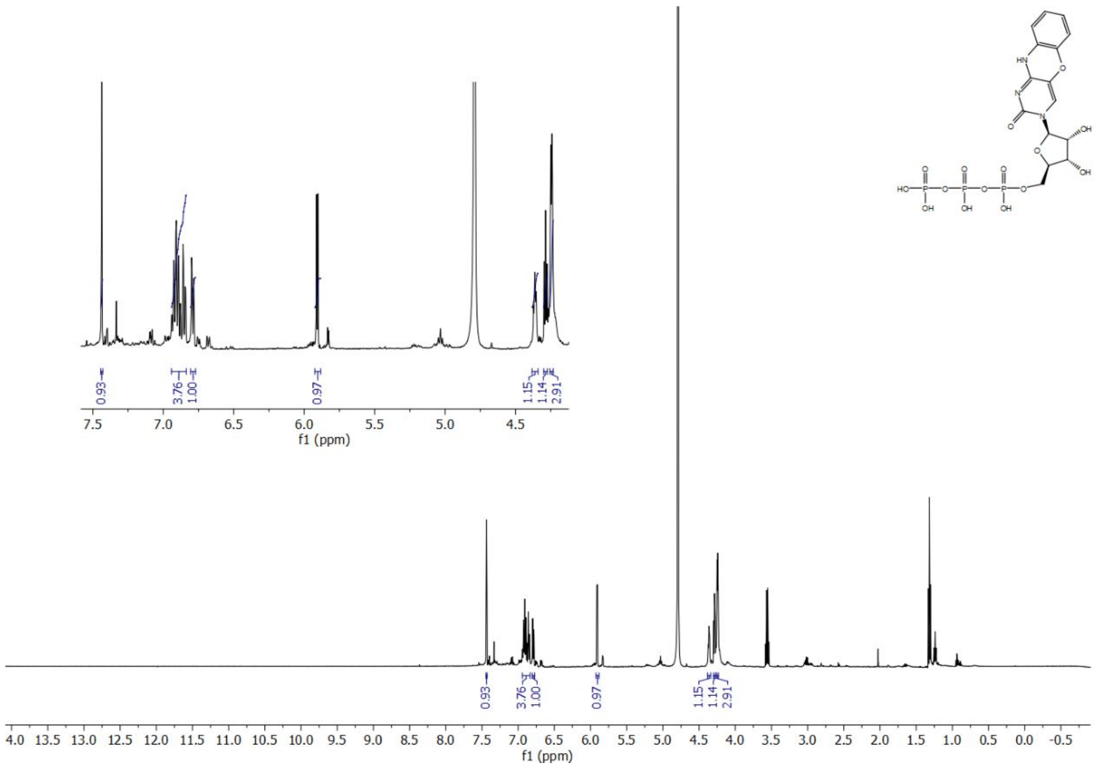

^{1}H spectrum after purification:

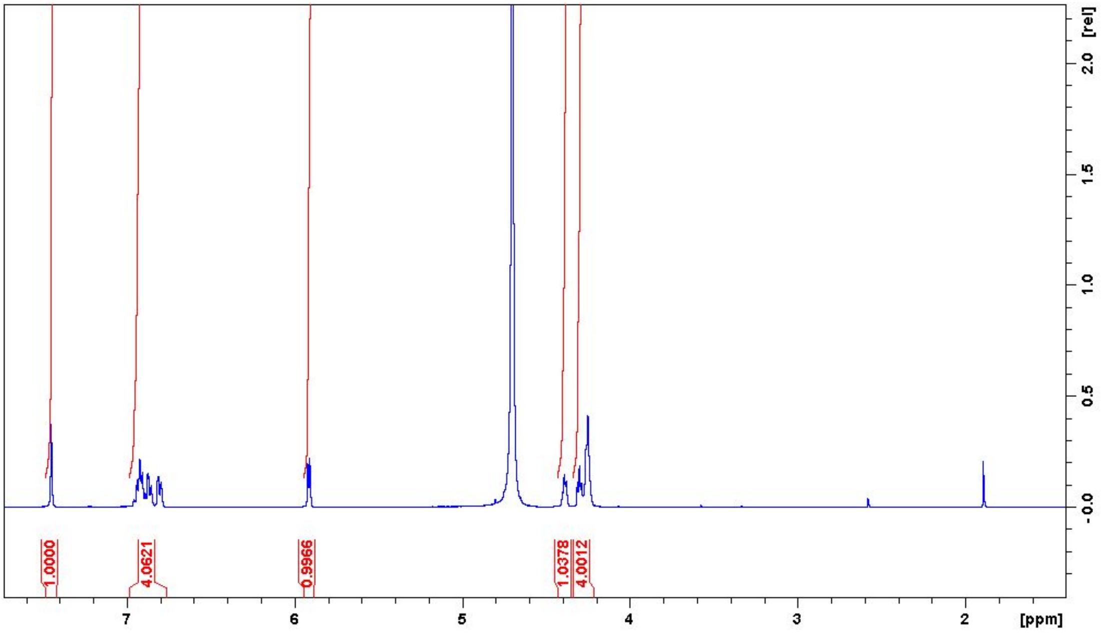

^{13}C NMR (126 MHz, D_2O) δ 155.8, 154.8, 142.4, 129.4, 124.9, 124.3, 122.3, 116.6, 88.8, 82.8, 73.4, 69.7, 64.5.

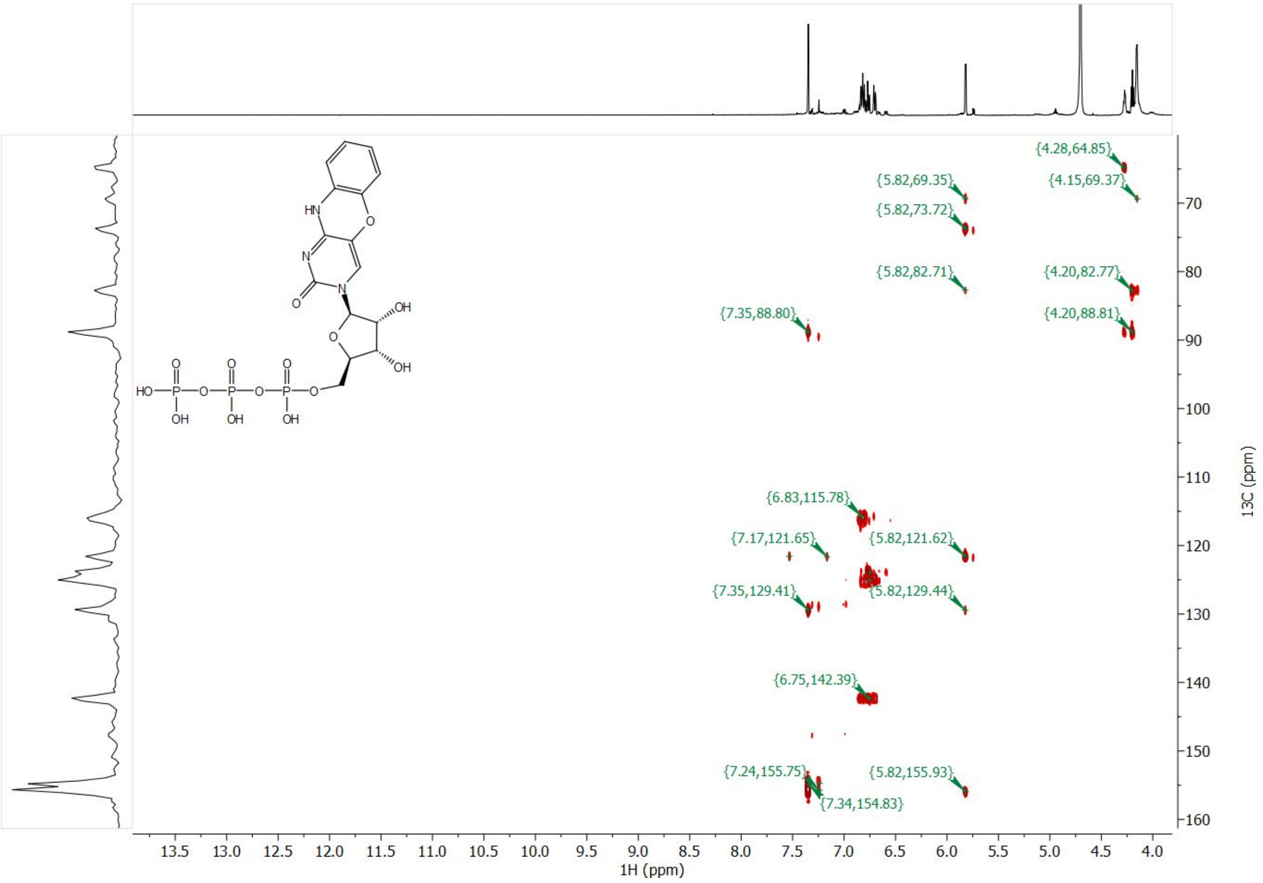

^{31}P NMR (202 MHz, D_2O) δ -10.89 (d, *J* = 18.5 Hz, 1P), -11.46 (d, *J* = 19.7 Hz, 1P), -23.21 (t, *J* = 19 Hz, 1P).

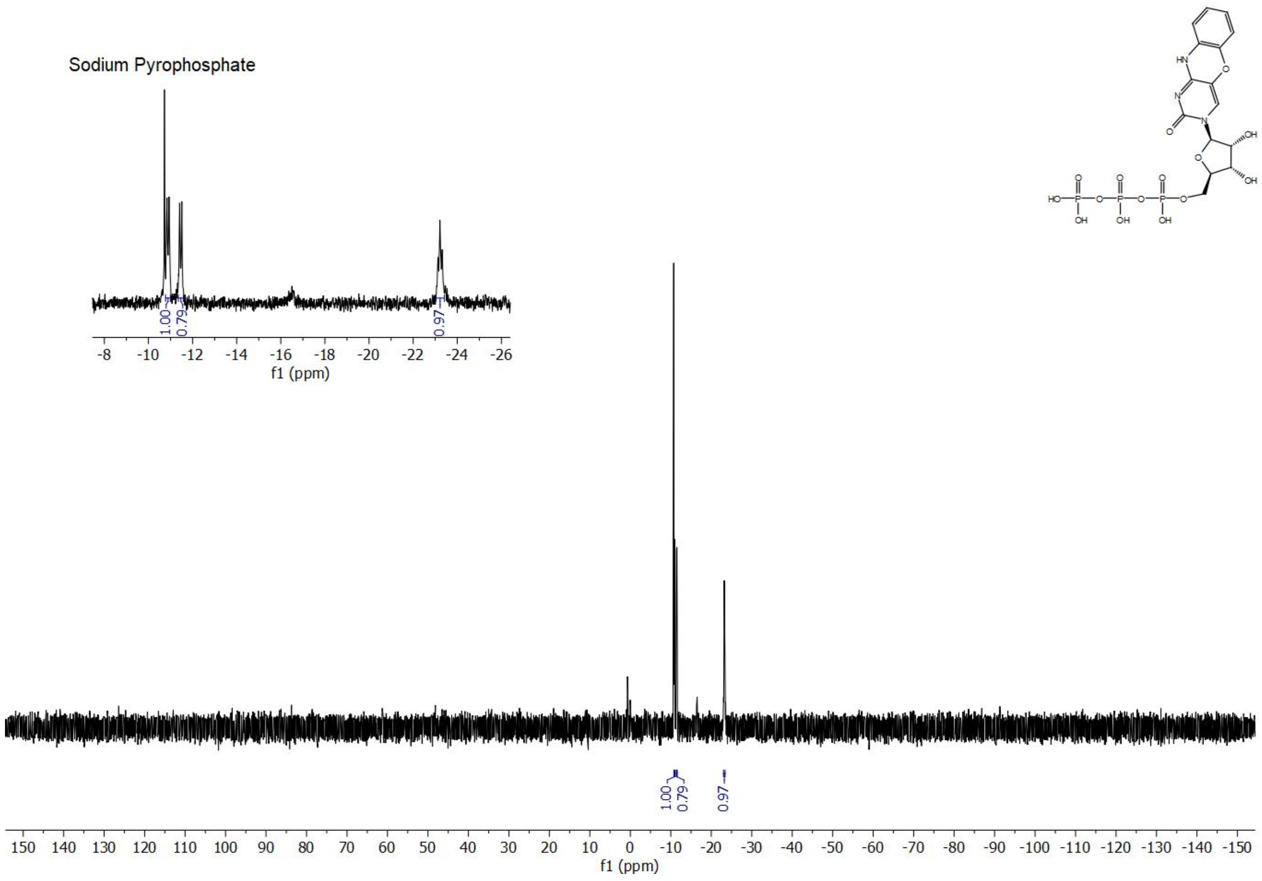

Purity analysis of tC^{O}-triphosphate **7** (Waters Acquity HSS T3 column, 2.1x50 mm, 0.4 mL/min, 2 to 99 % 50 mM NH_4OAc in water 80:20 EtOH):

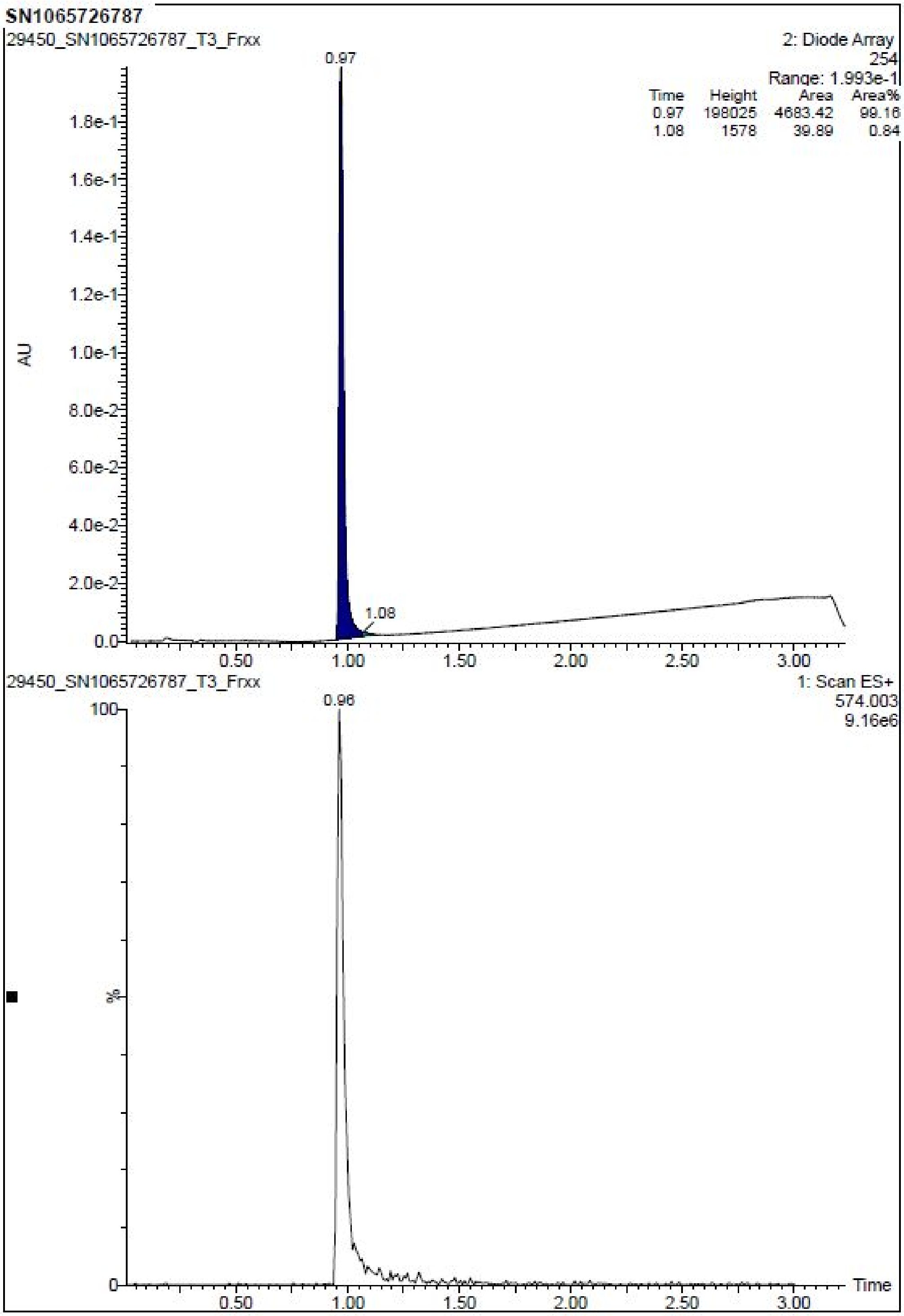

Experimental procedures: Spectroscopy

The tC^{O}-RNA products from the cell-free transcription reactions (prior to polyadenylation and capping, see Methods: Bio for details) were measured as received, *i.e.* in RNAse free Milli Q water. All measurements were carried out at room temperature (*ca.* 22°C) in a 3.0 mm path length quartz cuvette, with a sample volume of *ca.* 60 µL.

Steady state absorption. Absorption spectra were recorded on a Cary 5000 (Varian Technologies) spectrophotometer with a wavelength interval of 1.0 nm, integration time of 0.1 s, and a spectral band width (SBW) of 1 nm. All spectra were baseline corrected by subtracting the corresponding absorption from the solvent only. A second-order polynomial Savitzky-Golay (five points) smoothing filter was applied to all spectra. For samples exhibiting significant scattering, as evidenced by characteristic absorption in the long wavelength region (here for $\lambda > 475$ nm), an additional correction was applied. The scattering contribution ($A_{scatter}$) to the absorption was in such cases fitted (using absorption at 550–475 nm as input) to the Rayleigh scattering function (equation S1), where c is a proportionality constant and A_0 a constant, and then subtracted for all wavelengths.

$$A_{scatter}(\lambda) = log\left(\frac{1}{1 - c \times \lambda^{-4}}\right) + A_0 \qquad \text{(S1)}$$

Steady state emission. Emission spectra were recorded on a SPEX Fluorolog (Jobin Yvon Horiba) fluorimeter with excitation at 356 nm. Emission was collected at a right angle with an integration time of 0.1 s and wavelength interval of 1 nm. Monochromator slits were adjusted to achieve optimal signal output, leading to SBWs in the interval 1.5–2.5 nm on both the excitation and emission side. Emission spectra were corrected for Raman scattering from water by subtracting the corresponding emission from a sample containing only solvent. A second-order polynomial Savitzky-Golay (five points) smoothing filter was applied to all spectra.

Fluorescence quantum yield determination. Sample fluorescence quantum yields (Φ_F) were determined relative to a solution of quinine sulphate (Sigma) in 0.5 M H_2SO_4 ($\Phi_{F,REF}$ = 0.546)[4] and calculated according to equation S2.

$$\Phi_F = \Phi_{F,REF} \times \frac{\int_{\lambda_i}^{\lambda_f} I_S(\lambda) d\lambda}{\int_{\lambda_i}^{\lambda_f} I_{REF}(\lambda) d\lambda} \times \frac{A_{REF}}{A_s} \times \frac{\eta_S^2}{\eta_{REF}^2} \quad \text{(S2)}$$

Emission spectra for the sample, $I_S(\lambda)$, and reference, $I_{REF}(\lambda)$, were integrated between λ_i = 365 nm and λ_f = 700 nm. Absorption at the excitation wavelength (356 nm) for the sample (A_s) and reference (A_{REF}) were in the interval 0.05–0.11 for all samples. Adopted solvent refractive indices for the samples (water) and reference (0.5 M H_2SO_4) were η_S = 1.333 and η_{REF} = 1.339, respectively. All quantum yields are presented as mean ± standard deviation of two independent cell-free transcription reactions.

Time-resolved emission. Fluorescence lifetimes were determined using time-correlated single photon counting (TCSPC). Samples were excited using an LDH-P-C-375 (PicoQuant) pulsed laser diode with emission centered at 377 nm (FWHM pulse width was 1 nm and 70 ps with respect to wavelength and time, respectively), operated with a PDL 800-B (PicoQuant) laser driver at a repetition frequency of 10 MHz. Sample emission (458 nm, SBW = 10 nm) was collected at a right angle, through an emission polarizer set at 54.9° (magic angle detection). Photon counts were recorded on a R3809U-50 microchannel plate PMT (Hamamatsu) and fed into a LifeSpec multichannel analyzer (Edinburgh Analytical Instruments) with 2048 active channels (24.4 ps/channel), until the stop condition of 10^4 counts in the top channel was met. The instrument response function (IRF) was determined using a frosted glass (scattering) modular insert while observing the emission at 377 nm (SBW = 10 nm).

Fitting of fluorescence lifetimes. The intensity decays were fitted with IRF re-convolution to the multiexponential model shown in equation S3.

$$I(t) = \int_0^t IRF(t') \sum_{i=1}^{n} \alpha_i e^{-\frac{t-t'}{\tau_i}} dt' \quad \text{(S3)}$$

The least-square re-convolution fitting procedure was carried out using the DecayFit software (http://www.fluortools.com/software/decayfit). All decays were fitted to a tri-exponential (*n* = 3) model. The presented lifetimes are amplitude-weighted average lifetimes ($\bar{\tau}$), calculated using the pre-exponential factors (α_i) and lifetimes (τ_i) according to equation S4. The fitting parameters for the decays are shown in Supplementary Table 2.

$$\bar{\tau} = \sum_{i=1}^{n} \alpha_i \tau_i \quad \text{(S4)}$$

Cell-free transcription reaction kinetics. The ratio of the rate constants for cytosine *vs.* tC^O incorporation (k_C/k_{tC^0}) was calculated using the absorption spectra of the tC^O-RNA transcripts (A_{260} and A_{369}), and triphosphate initial concentrations ($[CTP]_0$ and $[tC^0TP]_0$) as input. Equations S5 and S6 follows upon assuming first-order reaction kinetics with respect to the triphosphate species [CTP] and [tC^OTP].

$$\frac{d[\text{CTP}]}{dt} = -k_C \times [\text{CTP}] \quad \text{(S5)}$$

$$\frac{d[tC^O\text{TP}]}{dt} = -k_{tC^0} \times [tC^O\text{TP}] \quad \text{(S6)}$$

Solving S5 and S6 for the respective rate constants renders equation S7, in which [C] and [tC^O] denote the concentration of *incorporated* C and tC^O, respectively.

$$\frac{k_C}{k_{tC^0}} = \frac{ln\left(\frac{[CTP]_0}{[CTP]_0 - [C]}\right)}{ln\left(\frac{[tC^0TP]_0}{[tC^0TP]_0 - [tC^O]}\right)} \quad \text{(S7)}$$

Using the Beer-Lambert law, absorption is related to nucleobase concentration according to equations S8 and S9. The following molar absorptivities (unit: $M^{-1}cm^{-1}$) were adopted: $\varepsilon_{260}^{tC^O}$ = 12200, ε_{260}^{C} = 7400,

ε_{260}^{G} = 11800, ε_{260}^{U} = 9300, ε_{260}^{A} = 15300, $\varepsilon_{369}^{tC^O}$ = 9370. It should be noted that the molar absorptivity of the tC^O adopted here ($\varepsilon_{260}^{tC^O}$= 12200 $M^{-1}cm^{-1}$) differs slightly from what has been published previously by our group (11000 $M^{-1}cm^{-1}$). This is due to a gravimetric re-evaluation of this parameter using a large amount of compound prior to starting this work.

$$A_{260} = 0.9 \times l \times (\varepsilon_{260}^{tC^O} \times [tC^0] + \varepsilon_{260}^{C} \times [C] + \varepsilon_{260}^{G} \times [G] + \varepsilon_{260}^{U} \times [U] + \varepsilon_{260}^{A} \times [A]) \quad \text{(S8)}$$

$$A_{369} = l \times \varepsilon_{369}^{tC^O} \times [tC^0] \quad \text{(S9)}$$

Assuming that the product RNA is uniform in size (1247 nucleotides), its base composition (A: 408, U: 272, G: 307, C: 260) allows for equations S10 – S13.

$$[tC^0] + [C] = [RNA] \times 260 \quad \text{(S10)}$$

$$[A] = [RNA] \times 408 \quad \text{(S11)}$$

$$[U] = [RNA] \times 272 \quad \text{(S12)}$$

$$[G] = [RNA] \times 307 \quad \text{(S13)}$$

Solving the equation system composed of S7 through S13 allows for quantification of k_C/k_{tC^0}, $[tC^0]$, $[RNA]$, $[C]$, $[A]$, $[U]$, and $[G]$. The average-strand tC^O incorporation degree (θ_{tC^O}) can then be calculated according to equation S14.

$$\theta_{tC^O} = \frac{[tC^0]}{[C] + [tC^0]} \quad \text{(S14)}$$

Using the volume of the cell-free reaction (50 µL) and resulting product solution (100 µL), equation S15 was applied to calculate the tC^0 incorporation yield (η_{tC^0}).

$$\eta_{tC^0} = \frac{[tC^0] \times 100\ \mu L}{[tC^0TP]_0 \times 50\ \mu L} \quad \text{(S15)}$$

Consonantly, the RNA yield (η_{RNA}) was calculated according to equation S16.

$$\eta_{RNA} = \frac{[RNA] \times 100\ \mu\text{L}}{[tC^0TP]_0 \times 50\ \mu\text{L}} \qquad \text{(S16)}$$

<u>Experimental procedures: Biochemistry</u>

The TdT, Taq, and Phusion polymerases were purchased from New England Biolabs as well as the natural dNTPs and NTPs. The AmpliScribe™ T7 High Yield Transcription Kit was purchased from Epicentre and the HiScribe T7 In Vitro Transcription Kit was obtained from New England Biolabs. The reverse transcriptase M-MLV RT as well as the T4 DNA ligase were obtained from Promega. The pGEM-T Vector kit was purchased from Promega. Acrylamide/bisacrylamide (29:1, 40 %) was obtained from Fisher Scientific. Visualization of PAGE gels was performed by fluorescence imaging using a Storm 860 or a Typhoon Trio phosphorimager with the ImageQuant software (GE Healthcare). Natural DNA and RNA oligonucleotides were purchased from Microsynth. Concentrations of short oligonucleotides were quantitated by UV spectroscopy using a UV5Nano spectrophotometer (Mettler Toledo).

General protocol for the TdT-mediated polymerization reactions

A solution containing 20 or 2 pmol of the fluorescently labeled ssRNA oligonucleotides (TdT1, TdT2, and TdT3), 1 U/µL of TdT, the modified nucleotide (200 µM final concentration), 1x reaction buffer (from Promega (containing 1 mM Co^{2+}) for Supplementary Fig. 1a and NEB for Supplementary Fig. 1b), metal cofactor (0.25 mM Co^{2+}, 1 mM Mn^{2+}, or 1 mM Mg^{2+} (final concentrations)) and H_2O (for a total reaction volume of 10 µL) was incubated at 37 °C for different reaction times. The reactions were then quenched by addition of 10 µL of loading buffer. The reaction products were then resolved by electrophoresis (PAGE 20 %) and visualized by phosphorimager analysis. Alternatively, products stemming from the TdT-

labeling of oligonucleotide TdT3 were irradiated with UV light prior and after purification with a NucleoSpin (Macherey-Nagel) clean-up kit.

In vitro transcription from dsDNA templates:

Buffer compositions**:**

1: buffer supplied from Epicentre in the AmpliScribe T7 High Yield Transcription Kit.

2: 40 mM Tris-acetate pH 8.0, 10 mM $Mg(OAc)_2$, 0.5 mM $MnCl_2$, 5 mM DTT, 0.1% Tween 20, 8 mM spermidine.[5]

3: 40 mM Tris·HCl, pH 8.0, 30 mM $MgCl_2$, 3.75 mM $MnCl_2$, 20 mM DTT, 0.01% Triton X-100, 3.6 mM spermidine.[6]

4: 40 mM Tris·HCl pH 8, 12 mM $MgCl_2$, 5 mM DTT, 0.01% Triton-X, 1 mM spermidine, 4% glycerol.[7]

5: 200 mM HEPES pH 7.5, 5.5 mM $MgCl_2$, 2 mM spermidine, 40 mM DTT, 0.01% Triton, 10% PEG8000, 1.5 mM $MnCl_2$, 10 U/ml yeast inorganic pyrophosphatase.[8]

6: 40 mM Tris·HCl pH 8, 8 mM $MgCl_2$, 2 mM spermidine, 25 mM NaCl.[9]

i) Transcription reaction with short dsDNA templates:

The T7 promoter (oligonucleotide T2) was hybridized with one of the DNA templates (B2, DNA3, DNA4) by heating at 75 °C for 3 min and then cooling down for 10 min on ice. The hybridized oligonucleotides were then added to a mixture containing 0.4 mM of each rNTP, RNAse inhibitor, DTT (10 mM final concentration for reactions with the AmpliScribe kit), reaction buffer, and RNAse free water and lastly 1.5 µL of T7 RNA polymerase (HiScribe T7 In Vitro Transcription Kit from New England Biolabs or AmpliScribe T7 High Yield Transcription Kit from Epicenter). The reaction mixtures were incubated for 1 h (Supplementary Fig. 2a) or 0.5 h (Supplementary Fig. 2b) at 37 °C and the reactions were quenched by

adding 2 µL EDTA 100 mM. The transcription products were precipitated by EtOH and then analyzed by gel electrophoresis (PAGE 20%) and imaged after staining with Midori green or silver.

ii) Transcription reactions with longer dsDNA templates:

DNA duplexes were prepared by PCR either using templates T12.3 or D1 Library[10] along with primers FWD-D1-RNA 2 and REV-D1-RNA 2. The PCR reaction mixture with template T12.3 consisted of 0.2 mM dNTPs, 1 µM of each primer, 2.6 ng/µL of template, 1x Ex Taq reaction buffer, and 20 U of Ex Taq DNA polymerase in a total volume of 800 µL. The PCR program started with a 5 min long denaturation step (95 °C) and was followed by 29 PCR cycles: 1. 30 sec at 95 °C, 2. 30 sec at 62 °C, 3. 1 min at 72 °C and a final elongation step (10 min at 72 °C). The PCR reaction mixture for the preparation of the dsDNA library consisted of 0.2 mM dNTPs, 1 µM of each primer, 72 nM template, 1x Thermopol reaction buffer, 1.5 mM $MgCl_2$, 0.5 mg/mL BSA, and 20 U of Taq DNA polymerase in a total volume of 800 µL. The PCR program started with a 5 min long denaturation step (95 °C) and was followed by 5 PCR cycles: 1. 1 min at 95 °C, 2. 1 min at 54 °C, 3. 1.5 min at 72 °C and a final elongation step (10 min at 72 °C). All PCR products were then purified by phenol/chloroform extraction followed by ethanol precipitation and filtration (10K Nanosep). All transcription reactions proceeded as described in section i) using reaction buffer 5. Gel analyses of the transcription reactions are highlighted in the manuscript (for D1 Library) and Supplementary Fig. 3 (for T12.3; see following paragraph).

Cloning and sequencing (RT-PCR stemming from template **T12.3):**

The products stemming from transcription reactions (both 30 min and 6 h reaction times) with CTP or **tC°TP** were reverse transcribed and then cloned and sequenced to assess the incorporation fidelity of the modified nucleotides during both transcription and reverse transcription.

i) reverse transcription:

20 pmol of the products (modified or natural) stemming from in vitro transcription reactions (see Section 4 and transcription reactions using template T12.3) are added to a reaction mixture containing 200 pmol primer REV-D1 128, the mixture is heated to 70 °C for 5 minutes then kept on ice for 10 seconds, then 0.2 mM dNTPs, RT reaction buffer, and 200 U of reverse transcriptase M-MLV RT are added in 50 µL final volume. The resulting reaction mixtures were then incubated at 42 °C for 1 hour. The resulting reverse transcripts were then purified by 10K Nanosep columns and DNA was quantified by UV absorbance. Gel analysis of products stemming from the transcription and reverse transcription reactions is shown in Supplementary Fig. 3.

ii) PCR amplification and A-tailing:

PCR reaction mixtures containing 0.1 pmol of the reverse transcription products, 0.5 µM primers **REV_D1 23** and **REV-D1 128**, 0.2 mM dNTPs, 0.02 U/µL of Phusion DNA polymerase, and Phusion reaction buffer were prepared for a total volume of 300 µL. After an initial denaturation step (98 °C for 30 seconds), 25 PCR cycles were carried out using the following program: 1. 98 °C for 10 seconds; 2. 54 °C for 30 seconds; 3. 72 °C for 20 seconds. A 5-minute final elongation step was also included in the program. After PCR, the reaction mixtures were purified using MiniElute purification kits (Qiagen) and subjected to A-tailing reactions. Briefly, 5 µL of the purified PCR mixtures were incubated for 30 min at 70°C in the presence of 1.5 mM $MgCl_2$, 0.2 mM ATP, 5U of Taq DNA polymerase, and reaction buffer 1x.

iii) Ligations and transformations:

Ligation reactions were carried out by preparing a reaction mixture containing 1 µL of the A-tailed PCR reaction products, 1 µL of the pGEM T vector (50 ng), T4 DNA ligase (6 Weiss U), and ligase reaction buffer in a total volume of 11 µL. The reaction mixtures were then incubated at room temperature under gentle stirring for 1 h. The resulting ligation products were then purified by filter dialysis (nitrocellulose 0.05 µM, Millipore). 3 µL of the ligation reactions were then added to 50 µL of beta 2033 competent cells

(frozen instead of freshly prepared). The cells were then subjected to electroporation at 1800V. After addition of 1 mL LB broth, the cells were incubated at 37 °C for 15 min. 100 µL of the mixtures were then streaked onto Petri dishes and centrifuged for 3 min at 8000 rpm. The supernatants were then removed and the resuspended pellets were streak onto LB-Carbenicillin (100 µg/ml final) / X-Gal (80 µg/mL) / IPTG (0.5 mM final) plates. The plates were then incubated at 37 °C for 12 h and white colonies were selected and grown overnight at 37 °C in LB containing carbenicillin medium (100 µg/mL), and plasmids were purified by use of a QIAprep Spin Miniprep Kit according to the manufacturer's instructions (QIAGEN). The selected plasmids were purified by use of a QIAprep Spin Miniprep Kit according to the manufacturer's instructions (QIAGEN). For the reaction products stemming from transcription with natural CTP, 41 plasmids were purified for the 30 min reaction products and 39 for the 6 h reaction time point. For the products with the modified triphosphate, 38 plasmids were purified for the 30 min reaction products and 39 for the 6 h reaction time point.

iv) Sequencing and analysis:

The sequencing (Illumina) of the purified plasmids was carried out using the Eurofins sequencing service with the sequencing primers M13 uni (-43) and M13 rev (-29) for the PCR amplification of the plasmids. Sequence alignment was performed with Benchling [Biology Software] (2018) using MAFFT Algorithm (https://benchling.com). Motif logos highlighted in the manuscript were made from these alignments using Weblogo 2.0.[11] The sequence alignments obtained (only shown for the products obtained with primer M13 uni (-43)) are shown in Supplementary Fig. 4 and 5.

Generation of H2B:GFP DNA template

The original coding sequence for H2B:GFP was taken from pCS2-H2B:GFP plasmid (Addgene, Plasmid #53744, Supplementary Fig. 6), manually codon-optimized to minimize the occurrence of poly-C_n stretches (n<3), in silico-assembled with an additional T7 promoter and other desired features (Shine-

Dalgarno/Kozak consensus sequences for enhancement of translation and a 3xStop, respectively at the 5' and 3' of the coding sequence itself, plus the needed HindIII/SnaBI restriction sites, to generate the ligation-prone sticky ends) and ordered from Twist Bioscience as a synthetic gene block (Supplementary Fig. 6). The obtained sequence was then PCR-amplified, using a Phusion Hot Start High-Fidelity Taq (Thermo Scientific), and subcloned into a HindIII/SnaBI-digested (Fast Digest enzymes, Thermo Scientific) empty pCS2 backbone. After ligation with T4 ligase for 1 h at room temperature (Roche), DH5α E. coli competent cells (Invitrogen) were transformed following the recommended protocol, and obtained colonies were screened by colony-PCR. The selected colony was then inoculated into a midiprep-scale volume of liquid Luria-Bertani growth medium (VWR) and plasmid DNA isolated using a PureLink Fast Low-Endotoxin Midi Plasmid Purification Kit (Thermo Scientific). The purified plasmid was finally digested again with HindIII/SnaBI and gel-purified, to generate the transcription template with the desired size.

Primers (Eurofins Genomics):

Twist-H2B.F: GAAGTGCCATTCCGCCTGAC

Twist-H2B.R: CACTGAGCCTCCACCTAGCC

H2B:GFP RNA transcription and purification

In vitro transcription reactions, for T7 and SP6 polymerases (Thermo Scientific), were assembled as recommended by the corresponding protocols, with few modifications that resulted in a consistently increased yield in all conditions:

5X Transcription buffer - 10 µl

NTP Mix, 10 mM each (2 mM final concentration) – volume depending on batch for tC^{O}TP

Linearized template DNA 1 µg – volume depending on concentration

RiboLock RNase Inhibitor - 1.25 µl (50 U)

T7/T3/SP6 RNA Polymerase - 3 µl (60 U, double compared to recommendations)

$MgCl_2$ 4 mM final concentration (increased as recommended by Thomen et al.)[12]

DEPC-treated Water qsp 50 µl

In vitro transcriptions were always performed at 20 °C for 14 h, then RNAs were purified using a Monarch RNA Cleanup kit (NEB), or homemade equivalent buffers and regenerated columns,[13] following the same rationale. It was possible to partially recover unreacted $tC^{O}TP$ from the transcription mixtures by HPLC to re-use for further assays. For cellular studies, each batch of RNA was then enzymatically added with a polyA tail (with a Poly(A) Polymerase, NEB protocol #M0276 with incubation extended to 1 hour) and a Cap 0 analog (using a Vaccinia capping system, NEB protocol #M2080), following the recommended procedures. This was necessary to increase, respectively, affinity for the cellular translation machinery and resistance to endogenous nucleases.

Denaturing bleach-agarose gels

For a qualitative check of all in vitro synthesized RNAs, a denaturing agarose gel was run, in presence of 1,5 % bleach (Sigma Aldrich), as recommended in Aranda et al.[14] RNAs were first mixed with a 6x DNA loading dye (Invitrogen) and then heat-denatured at 70°C for 10 min in a heating block, then immediately transferred and kept on ice. The RiboRuler High Range RNA Ladder (Thermo Scientific) underwent the same treatment; 2 µl of RNA ladder were loaded along the samples and the gel was run at constant voltage (70 V) for 1 h and then imaged, under UV transillumination (302 nm) using a ChemiDoc Touch (BioRad). To counterstain the whole gel, and especially the lanes without $tC^{O}TP$-containing samples, a standard ethidium bromide staining was finally performed at room temperature for 10 min and gentle rocking, followed by two washes in TAE and then a final wash in distilled water (10 min each).

Cell culture

Human neuroblastoma SH-SY5Y cells (Sigma-Aldrich) were grown in a 1:1 mixture of minimal essential medium (HyClone) and nutrient mixture F-12 Ham (Sigma-Aldrich) supplemented with 10% fetal bovine serum (FBS), 1% non-essential amino acids (Lonza) and 2 mM L-glutamine. For the tracking experiments, an in-house generated model of human hepatic Huh-7 cells stably overexpressing mRFP-Rab5 were cultured in DMEM/GlutaMax/High glucose (Gibco) supplemented with 10 % FBS. The cells are detached with trypsin-EDTA 0.05 % (Gibco) and passaged twice a week.

Electroporation or chemical transfection

Cells were electroporated either with 9.7 µg of $tC^{O}TP$ (for in vitro incorporation experiments) or 100 ng of tC^{O}-labeled mRNA per 10^5 cells (for in vitro translation, cytotoxicity assessment, flow cytometry analysis and confocal microscopy), using a Neon Transfection System (Invitrogen, Carlsbad, CA, US) and following the protocol for 10 µL Neon Tip provided by the manufacturer, with a triple pulse of 1200 V and a pulse width of 20 ms. For chemical transfection, SH-SY5Y cells were seeded one day prior transfection at a density of 0.8 10^6 cells/mL, in 48-well plate or glass-bottomed culture dishes for flow cytometry of confocal microscopy analysis, respectively. Lipofectamine MessengerMAX was used as chemical reagent for transfection according to the manufacturer's instructions. Briefly, the reagent was diluted and incubated for 10 min at room temperature in Opti-MEM medium. The tC^{O}-mRNA constructs were added to the reagent to reach a 1:1 final ratio reagent-mRNA (v/w), followed by a 5 min incubation at room temperature allowing the complex mRNA-lipid to form. Cells were incubated with this complex up to 72 h. To address the impact of the dye incorporation on RNA translation, SH-SY5Y cells were electroporated or chemical transfected with commercially available non-labeled (NL) or Cyanine5-labeled (Cy5) eGFP encoding mRNAs (Trilink®) has described here.

Cytotoxicity assessment

Cell membrane integrity was determined using the Pierce™ LDH Cytotoxicity Assay Kit (Invitrogen) according to the manufacturer's instructions. Briefly, LDH released in the supernatants of cells 24 h post-electroporated or post-transfected with tC^{O}-labeled mRNA, or Cy5-mRNA, was measured with a coupled enzymatic assay which results in the conversion of a tetrazolium salt into a red formazan product. The absorbance was recorded at 490 nm and 680 nm. The toxicity was expressed as the percentage of LDH release in supernatant compared to maximum LDH release (supernatant + cell lysate). Data are means ± SD from three experiments performed in triplicate.

Flow cytometry

Following electroporation of tC^{O}-labeled mRNA, cells were seeded in 48-well plate (2.10^5 cells/well) and the expression of H2B:GFP in cells was quantified by flow cytometry. Briefly, 24 h, 48 h or 72 h post-electroporation or post-transfection with tC^{O}-mRNA, non-labeled mRNA or Cy5-mRNA, cells were harvested and analyzed on a Guava EasyCyte 8HT flow cytometer (Millipore). Data are mean fluorescence intensities ± SD of gated single living cells from three experiments performed in triplicate. The average fluorescence intensities were baseline corrected by subtracting the signal for RNase-free water electroporated or transfected cells. All flow cytometry data were analyzed in Flowing software (version 2.5.1) and displayed using R (http://www.R-project.org/).[15]

H2B:GFP: Excitation 488 nm; Emission 525-530 nm.

Confocal microscopy

After electroporation, cells were seeded in glass-bottomed culture dishes (MatTek glass-bottomed or in 4-sectors subdivided CELLview dishes; 2.10^5 cells/chamber). For tracking experiment, the Huh-7 cells stably overexpressing mRFP-Rab5 were incubated with lipofectamine/tC^{O}-mRNA complex and time-lapse was recorded up to 20 h post-chemical transfection. Confocal images were acquired on a Nikon C2+

confocal microscope equipped with a C2-DUVB GaAsP Detector Unit and using an oil-immersion 60 × 1.4 Nikon APO objective (Nikon Instruments, Amsterdam, Netherlands). Data were processed with the Fiji software.[16]

H2B:GFP: Excitation 488 nm; Emission 495-558 nm.

tC^{O}-labeled mRNA: Exc. 405 nm; Em. 447-486 nm.

Cy5-labeled mRNA: Exc. 640 nm; Em. 652-700 nm.

mRFP-Rab5: Exc. 561 nm; Em. 565-720 nm.

Cell-free translation

Cell-free translation reactions were performed using E. coli bacterial lysates and an Expressway™ Mini Cell-Free Expression System (Thermo Scientific). Calmodulin-like 3 protein is provided as a positive control plasmid (pEXP5-NT/CALML3) in the kit itself; this DNA vector contains a T7 polymerase promoter and a 6xHis tag, hence it was first in vitro-transcribed in presence of the desired concentrations of $tC^{O}TP$ (*vide supra*). The obtained RNAs, once purified, were used as templates for the cell-free translation reaction according to the manufacturer's recommendations:

E. coli slyD- Extract - 20 µl

2.5X IVPS E. coli Reaction Buffer (-A.A.) - 20 µl

50 mM Amino Acids (-Met) - 1.25 µl

75 mM Methionine* - 1 µl

T7 Enzyme Mix - 1 µl (omitted when using tC^{O}-labeled RNAs)

DNA Template - 1 µg (when testing the tC^{O}-labeled RNAs, added the same amount of RNA instead)

DNase/RNase-free distilled water qsp 50 µl

Coomassie staining and Western Blots

Protein samples from in vitro translation experiments were quantified with a Qubit Protein Assay kit (Thermo Scientific), mixed with 6x SDS Laemmli reducing buffer (Alfa Aesar), then heat-denatured at 85 °C for 10 min and kept at room temperature until needed. Samples were generally run in 1 mm polyacrylamide 4-20 % Novex MES/SDS gels (Thermo Scientific) and using a Mini Gel Tank, with the PSU set at constant voltage (200 V). For Coomassie staining, the gel was then washed three times in boiling water, to remove excess of SDS, on a benchtop shaker; a 1x Coomassie non-toxic staining solution was added to the gel and microwaved until initial boiling.[17] Gel was finally washed after the appropriate incubation time, to remove excess of background noise, in distilled water and imaged using a ChemiDoc Touch. For Western Blot, the gels were blotted onto PVDF LF ethanol-activated membranes (BioRad) with a TransBlot semi-dry system (BioRad), according to manufacturer's recommendations (settings for 1mm-thick gels and mixed weight proteins). PVDF membranes were then washed 5 min in TBS-T (TBS and 0,1 % Tween-20, Sigma Aldrich), blocked in 5 % milk in TBS for 1 h at room temperature and incubated with the appropriate primary antibody dilutions. After 3x5 min washes in TBST and an incubation of 1 h with the corresponding HRP-conjugated secondary antibodies, the membrane was washed again three times in TBS-T, once in TBS and once more in distilled water. Finally, membranes were incubated with a minimal volume of SuperSignal West Pico PLUS (Thermo Scientific) and imaged with a ChemiDoc Touch. Primary antibodies: mouse monoclonal anti-6xHistidine tag (Invitrogen) and mouse monoclonal anti-GAPDH (ref. 437000, Invitrogen), both diluted 1:1000 in 3 % BSA/TBS-T. Secondary antibodies: HRP-conjugated polyclonal goat anti-Ms (ref. A16072, Invitrogen), used at 1:10000 dilution in TBS-T.

Name	Sequence (5’ to 3’)[a]
**T2**	TAA TAC GAC TCA CTA TAG
**B2**	ATT ATG CTG AGT GAT ATC CTG TAC TCC TAA TGG GTA CA
**DNA 3**	ATT ATG CTG AGT GAT ATC CTT CTC CTT CAC TCC TCT C
**DNA 4**	ATT ATG CTG AGT GAT ATC CTA CTC CTT CCT TCC TCT C
**T12.3**	GCC TGT TGT GAG CCT CCT AAC GCA CCG GTC GCA GGT TTA GCA AGG TCA CAA GCT GGC ATC AAG GTT GCC CCA TGC TTA TTC TTG TCT CCC
**D1 Library**	GCC TGT TGT GAG CCT CCT AAC N_{49} CA TGC TTA TTC TTG TCT CCC
**FWD-D1-RNA 2**	TAA TAC GAC TCA CTA TAG GGA GAC AAG AAT AAG CAT G
**REV-D1-RNA 2**	GCC TGT TGT GAG CCT CCT AAC
**FWD-D1-RNA 1**	TAA TAC GAC TCA CTA TAG GCC TGT TGT GAG CCT CCT AAC
**REV-D1-RNA 1**	GGG AGA CAA GAA TAA GCA TG
**REV-D1 128**	GCC TGT TGT GAG CCT CCT AAC
**REV_D1 23**	GGG AGA CAA GAA TAA GCA TG
**M13 uni (-43)**	AGG GTT TTC CCA GTC ACG ACG TT
**M13 rev (-29)**	CAG GAA ACA GCT ATG ACC
**TdT1**	FAM-GCA AGC ACA GAC AUC AG
**TdT2**	FAM-GGG AAG UGC UAC CAC AAC UUU AGC CAU AAU GUC ACU UCU GCC GCG GGC AU
**TdT3[b]**	GGG AAG UGC UAC CAC AAC UUU AGC CAU AAU GUC ACU UCU GCC GCG GGC AUG CGG CCA GCC A

[a] the T7 promoter sequence is underlined; [b] obtained by transcription reaction.

Supplementary Table 1. Oligonucleotide sequences.

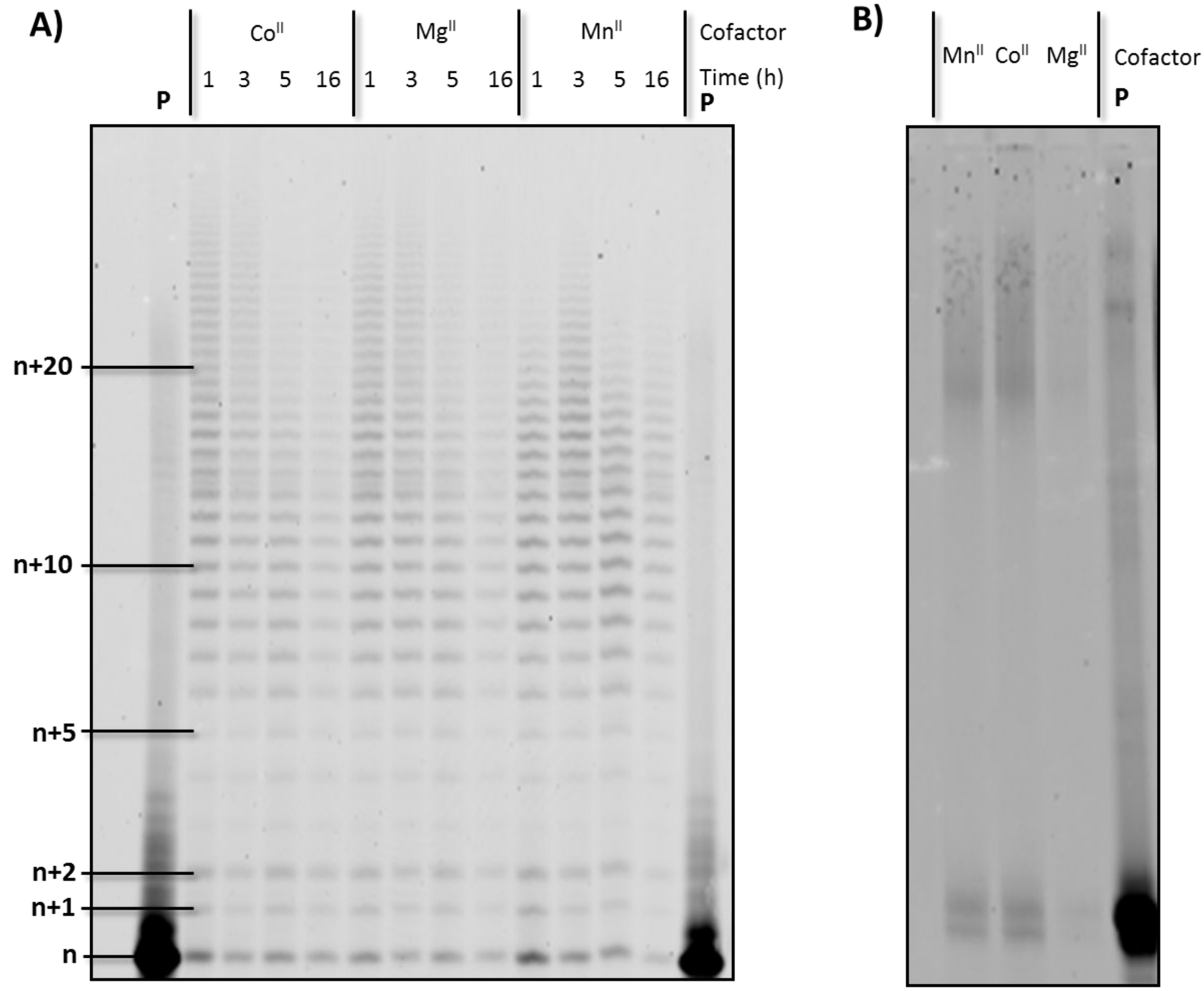

Supplementary Figure 1. Gel images (PAGE 20%) of the products stemming from the TdT-mediated tailing reactions with the modified triphosphate carried out using A) primer **TdT1** (also shown in the manuscript) and B) **TdT2**, different metal cofactors and reaction times. P indicates the unreacted primer.

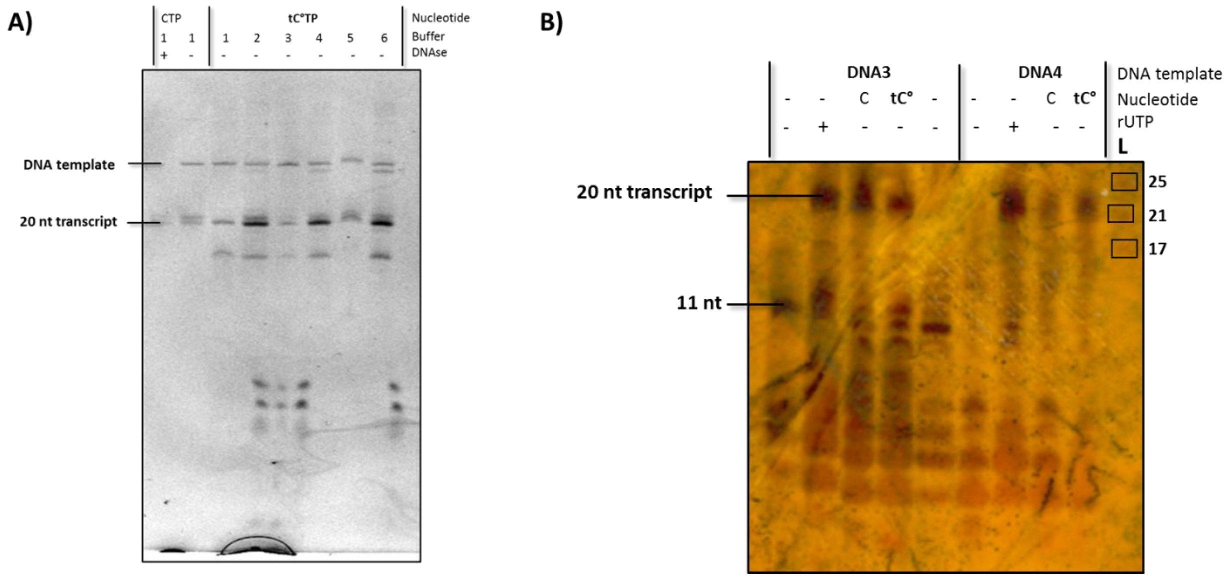

Supplementary Figure 2. Gel images (PAGE 20%) of transcription reactions with CTP and tC°TP. A) reactions performed with DNA oligonucleotides B2 and T2 and different buffer conditions (see Methods for buffer compositions). Midori green was used for visualization. B) transcription reactions with DNA templates DNA3 and DNA4. Control reactions were performed in the presence or absence of rUTP and all reactions were carried out in buffer 5 at 37°C for 30 min. L represents a ladder and visualization was performed by silver staining.

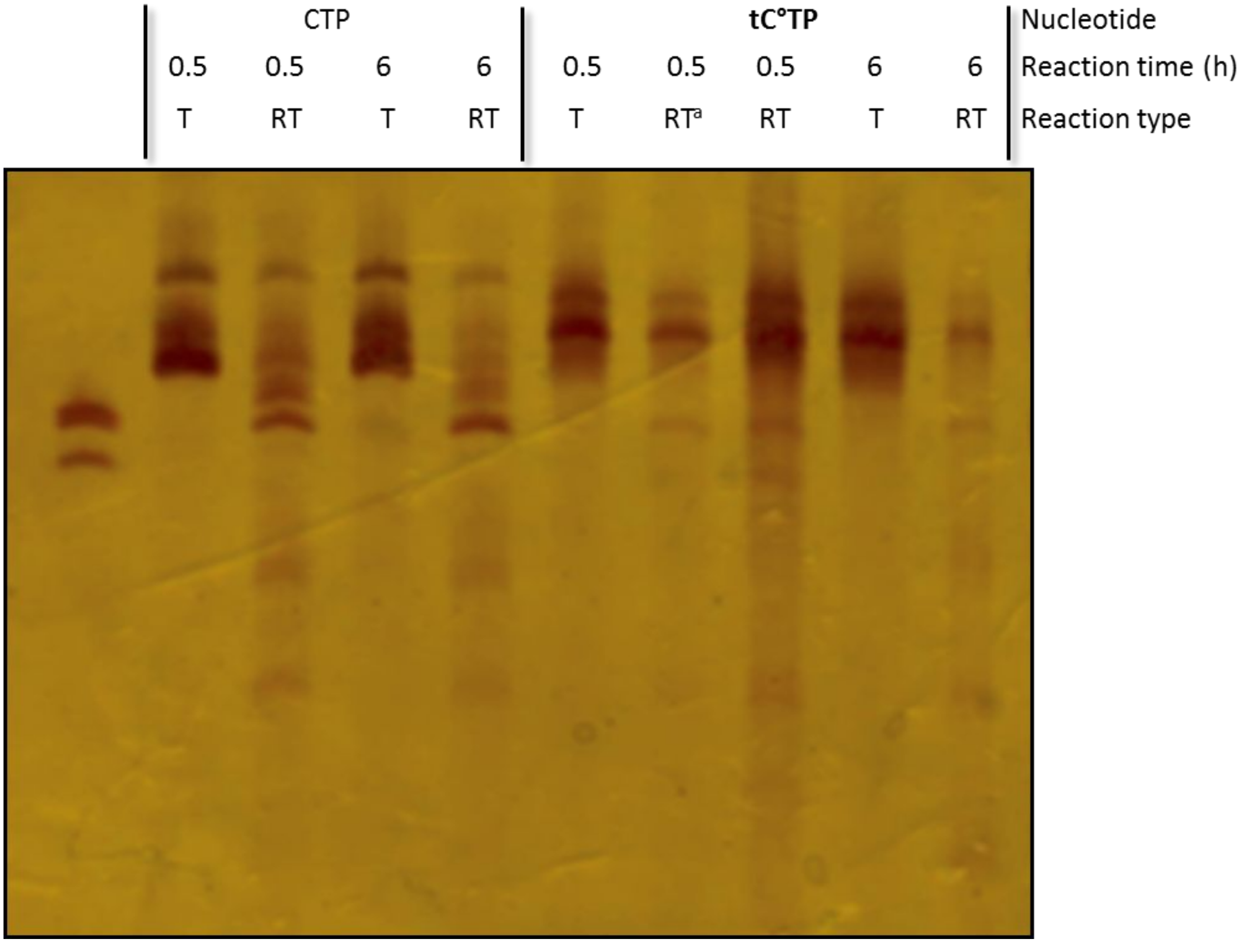

Supplementary Figure 3. Gel analysis (PAGE 10%) of the products stemming from transcription (T) from the T12.3 DNA template and subsequent reverse transcription (RT) reactions. The reaction times (0.5 and 6 h) refer to the times used for the transcription reactions. The leftmost lane shows the initial T12.3 DNA template. Visualization by silver staining. [a]5x less material was loaded on the gel.

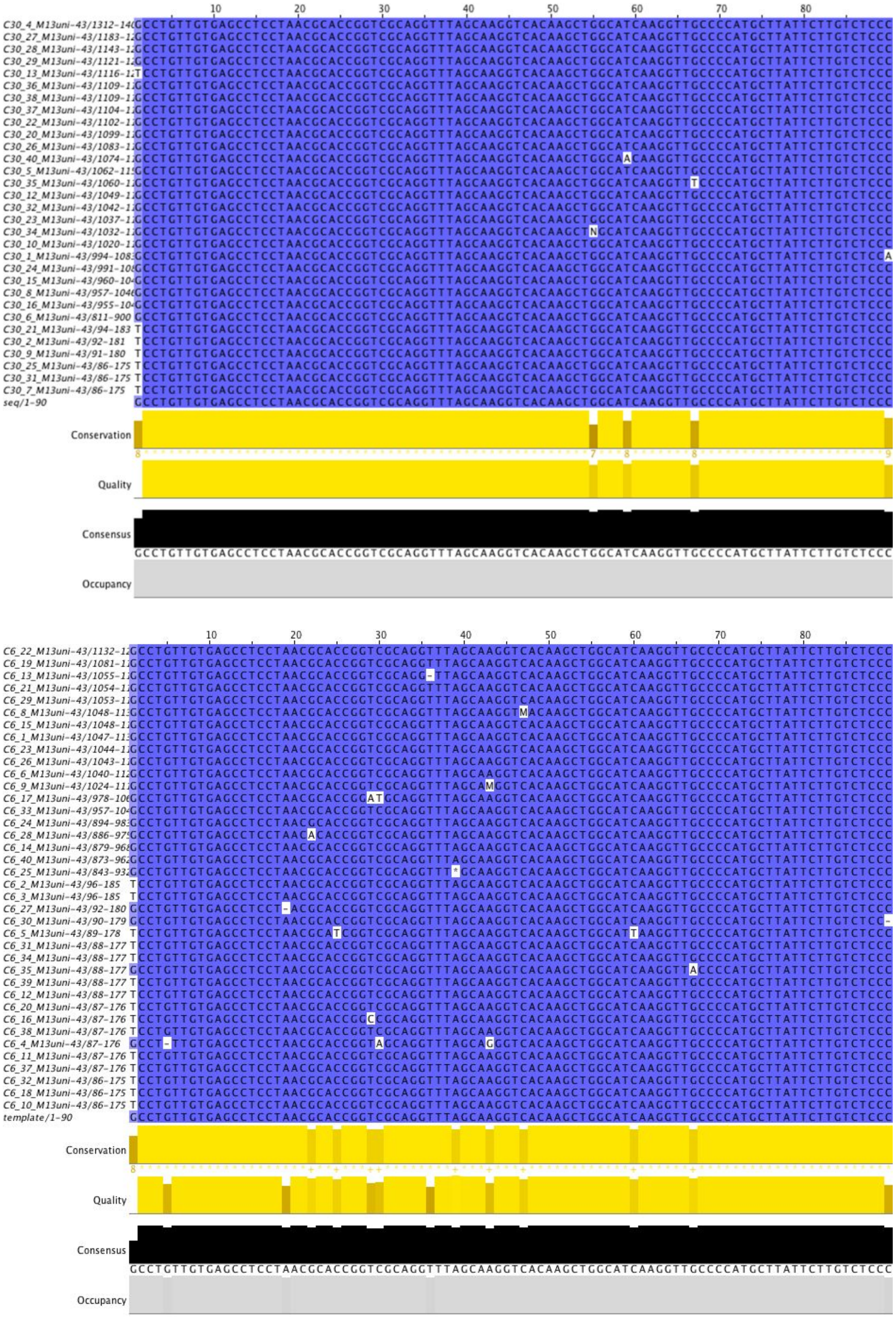

Supplementary Figure 4. Multiple alignment of the sequences stemming from the transcription reactions with CTP (up: 30 min and down: 6 h reaction time). Y: C or T, S: G or C, R: A or G, W: A or T, K: G or T, M: A or C, N: any nucleotide.

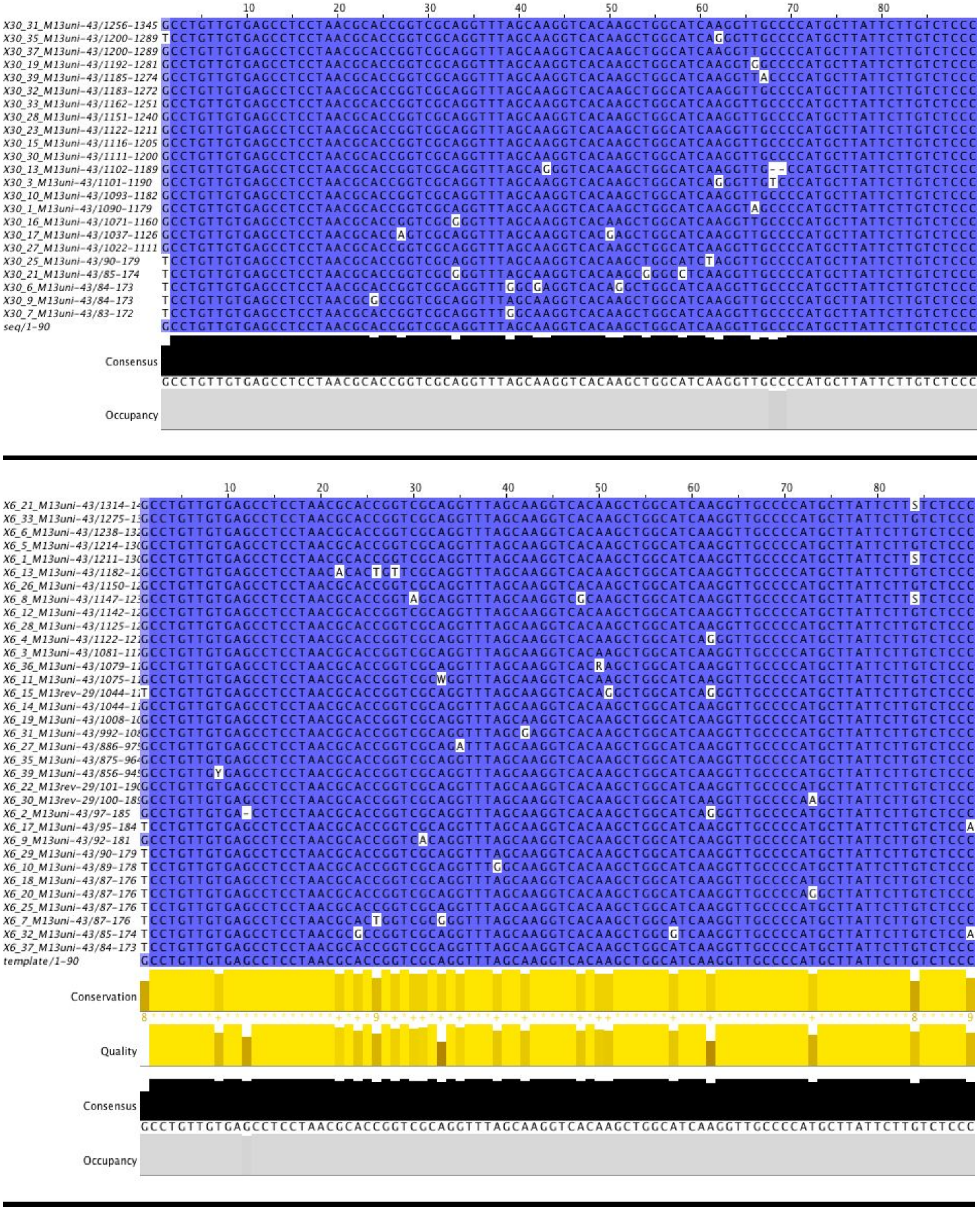

Supplementary Figure 5. Multiple alignment of the sequences stemming from the transcription reactions with tC°TP (up: 30 min and down: 6 h reaction time). Y: C or T, S: G or C, R: A or G, W: A or T, K: G or T, M: A or C, N: any nucleotide.

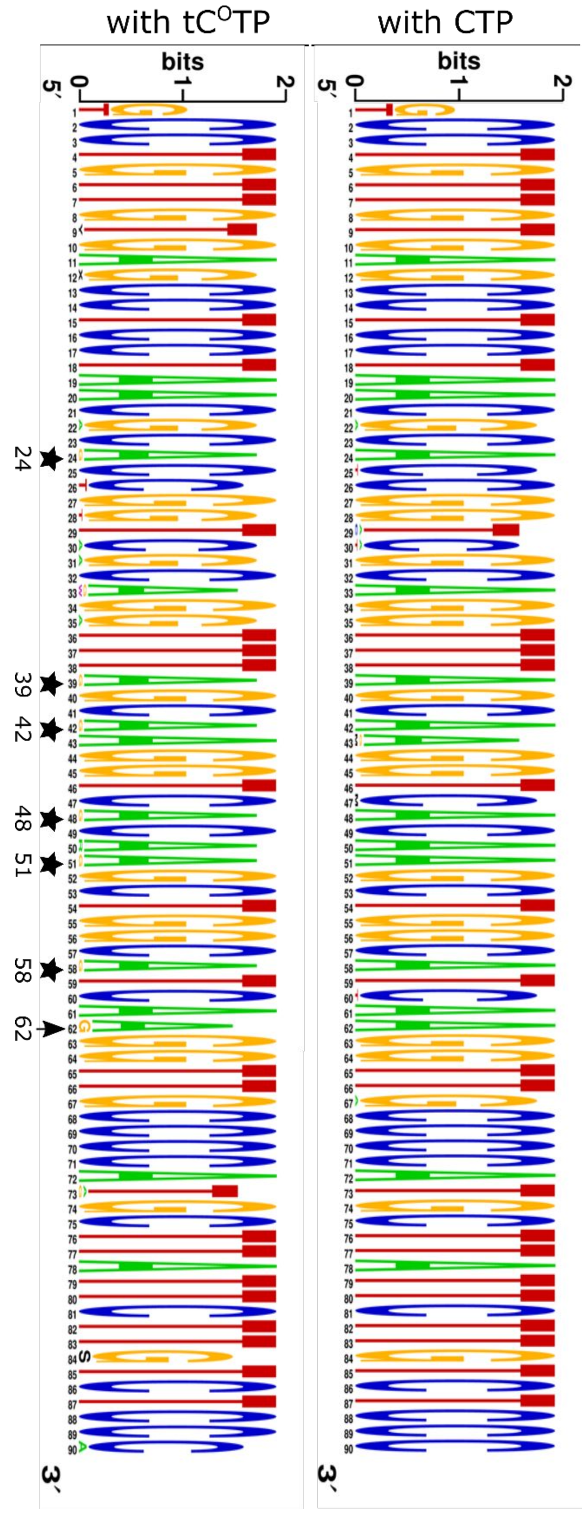

Supplementary Figure 6. Sequence logo from the cloning-sequencing protocol of reverse transcription products from the modified T12.3 RNA (right: products from a 6 h transcription reaction with CTP; left: products from a 6 h transcription reaction with $tC^{O}TP$). Lower (stars) and higher (arrow) frequency A to G transversions are highlighted.

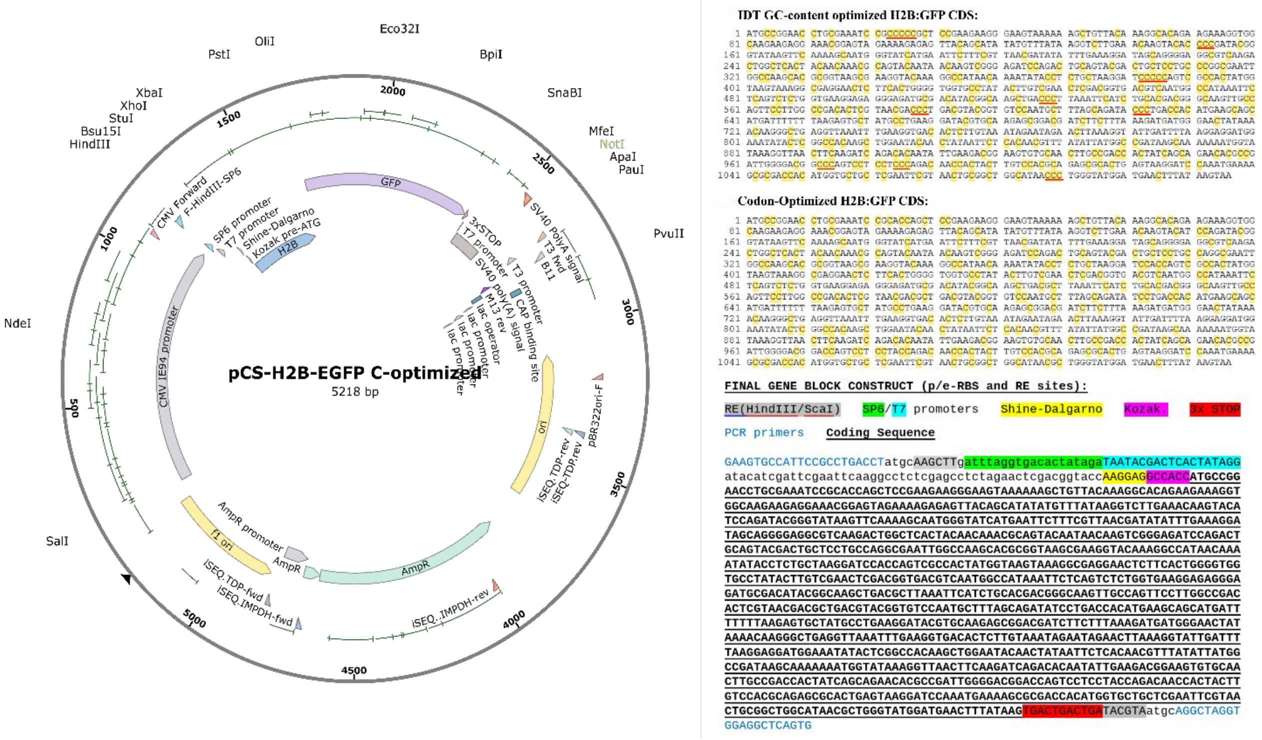

Supplementary Figure 7. pCS2-H2B:GFP plasmid, optimization of the H2B:GFP template and final construct.

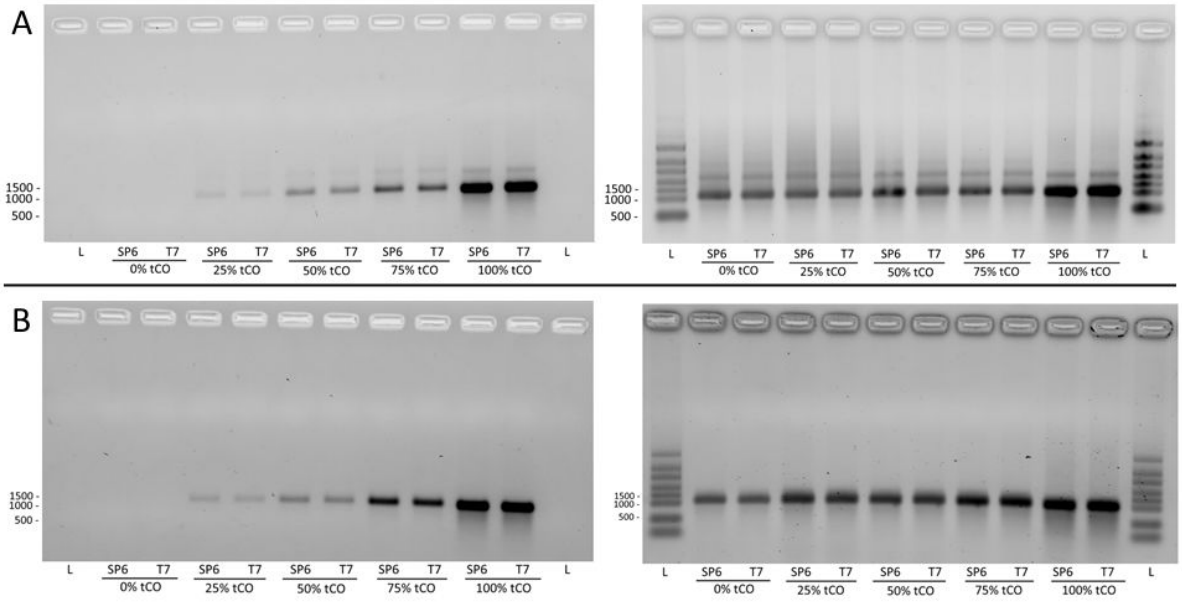

Supplementary Figure 8. Incorporation of tC^{O} into full length mRNA by SP6 and T7 RNA polymerase assisted in vitro transcription. (a) Denaturing agarose bleach gels showing RNA transcripts formed at five different $tC^{O}TP/CTP$ ratios (0-100%). The produced RNA was visualized directly by tC^{O} fluorescence (left image) and after ethidium bromide staining (right image). The RNA samples were heat-denatured (65 °C for 5 min, 1.5 % bleach in the gel) prior to loading on the gel. (b) Denaturing bleach gels of the same RNA transcripts as in (A) but at stronger denaturing conditions (70 °C for 10 min., 2 % bleach in the gel). The RiboRuler High Range RNA ladder was used.

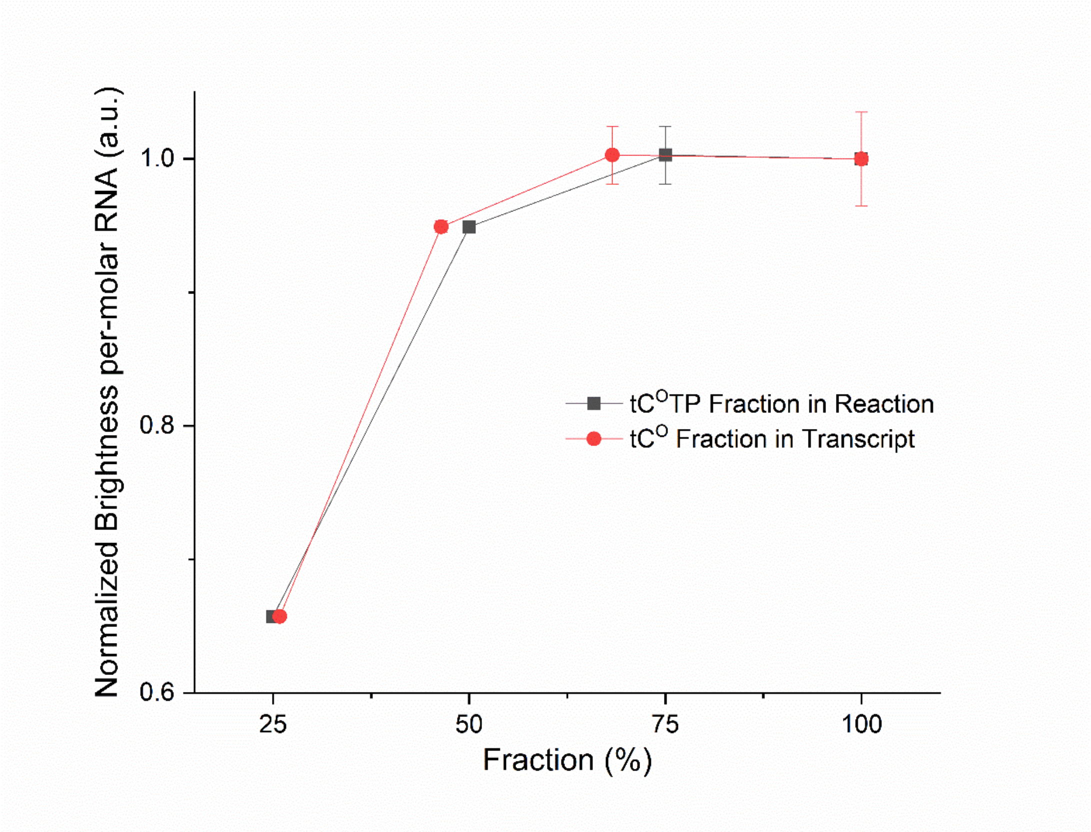

Supplementary Figure 9. Normalized per-molar RNA strand brightness of the tC^{O}-modified transcripts at 369 nm. The brightness was obtained by multiplying the fluorescence quantum yield (figure 4d) with the normalized absorption at 369 nm (figure a). The values were then normalized to 1 at the 100% fraction. Error bars reflect the standard deviations from the quantum yield determination.

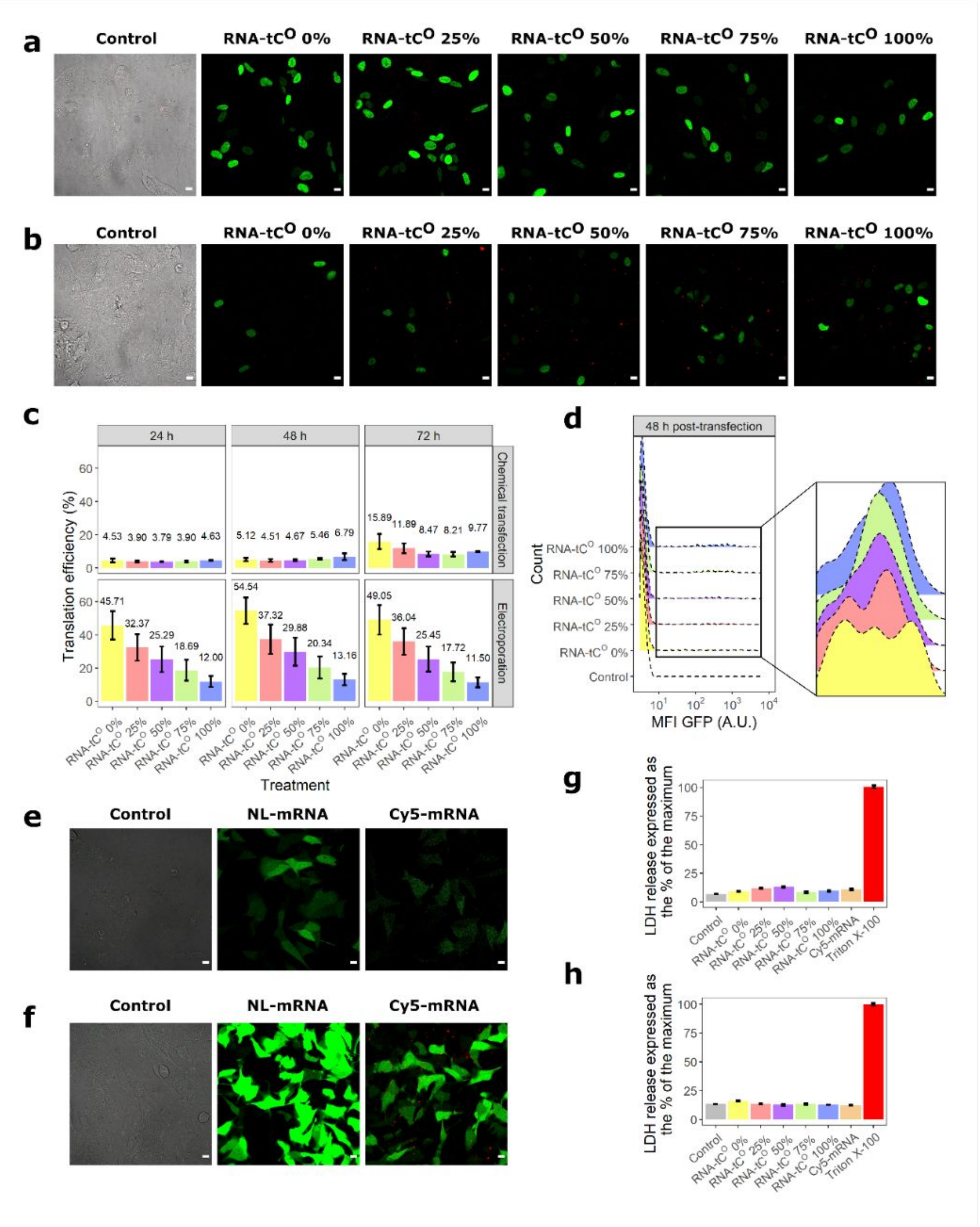

Supplementary Figure 10. Translation efficiency of the modified RNA constructs in human cells and cytotoxicity assessment. Representative confocal images (large view, scale bar: 10 µm) of RNA-tC^{O} constructs and mRNAs from Trillink® transfected by (a, e) electroporation or (b, f) chemical transfection. (c) Percentages of positive cells for H2B:GFP at 24 h, 48 h and 72 h post-transfection with RNA-tC^{O} constructs. (d) Representative histogram of the GFP signal distribution in single living cells at 48 h post-chemical transfection. Cytotoxicity assessment performed 24 h (g) post-electroporation or (h) post-chemical transfection using the LDH cell membrane integrity assay.

	transcript	A_1	T_1 (ns)	A_2	T_2 (ns)	A_3	T_3 (ns)	$\bar{\tau}$ (ns)	X^2
SET 1	25%	0.19	0.67	0.49	3.2	0.31	5.6	3.5	1.09
	50%	0.28	0.71	0.43	2.8	0.30	5.2	2.9	1.06
	75%	0.33	0.68	0.45	2.6	0.23	5.2	2.5	1.12
	100%	0.33	0.48	0.44	2.1	0.23	4.8	2.2	1.00
SET 2	25%	0.21	0.63	0.40	2.8	0.34	5.3	3.3	0.99
	50%	0.26	0.64	0.41	2.7	0.30	5.2	3.0	1.03
	75%	0.29	0.50	0.42	2.1	0.22	4.8	2.4	1.02
	100%	0.37	0.47	0.40	1.9	0.39	4.5	2.0	1.01

Supplementary Table 2. Fitted lifetime parameters for the TCSPC experiments. The X^2-value (Chi-Square) was evaluated to indicate goodness of fit.

References

1. Füchtbauer, A. F.; Preus, S.; Börjesson, K.; McPhee, S. A.; Lilley, D. M. J.; Wilhelmsson, L. M., Fluorescent RNA cytosine analogue – an internal probe for detailed structure and dynamics investigations. *Sci. Rep.* **2017,** *7* (1), 2393.
2. Ducho, C.; Görbig, U.; Jessel, S.; Gisch, N.; Balzarini, J.; Meier, C., Bis-cycloSal-d4T-monophosphates: Drugs That Deliver Two Molecules of Bioactive Nucleotides. *J. Med. Chem.* **2007,** *50* (6), 1335-1346.
3. Warnecke, S.; Meier, C., Synthesis of Nucleoside Di- and Triphosphates and Dinucleoside Polyphosphates with cycloSal-Nucleotides. *J. Org. Chem.* **2009,** *74* (8), 3024-3030.
4. Eaton, D. F., Reference materials for fluorescence measurement. In *Pure and Applied Chemistry*, 1988; Vol. 60, p 1107.
5. Sousa, R., [4] Use of T7 RNA polymerase and its mutants for incorporation of nucleoside analogs into RNA. In *Methods in Enzymology*, Academic Press: 2000; Vol. 317, pp 65-74.
6. Trevino, S. G.; Zhang, N.; Elenko, M. P.; Lupták, A.; Szostak, J. W., Evolution of functional nucleic acids in the presence of nonheritable backbone heterogeneity. *Proceedings of the National Academy of Sciences* **2011,** *108* (33), 13492.
7. Vaught, J. D.; Dewey, T.; Eaton, B. E., T7 RNA Polymerase Transcription with 5-Position Modified UTP Derivatives. *J. Am. Chem. Soc.* **2004,** *126* (36), 11231-11237.
8. Meyer, A. J.; Garry, D. J.; Hall, B.; Byrom, M. M.; McDonald, H. G.; Yang, X.; Yin, Y. W.; Ellington, A. D., Transcription yield of fully 2′-modified RNA can be increased by the addition of thermostabilizing mutations to T7 RNA polymerase mutants. *Nucleic Acids Res.* **2015,** *43* (15), 7480-7488.
9. Stengel, G.; Urban, M.; Purse, B. W.; Kuchta, R. D., Incorporation of the Fluorescent Ribonucleotide Analogue tCTP by T7 RNA Polymerase. *Anal. Chem.* **2010,** *82* (3), 1082-1089.
10. Binning, J. M.; Wang, T.; Luthra, P.; Shabman, R. S.; Borek, D. M.; Liu, G.; Xu, W.; Leung, D. W.; Basler, C. F.; Amarasinghe, G. K., Development of RNA Aptamers Targeting Ebola Virus VP35. *Biochemistry* **2013,** *52* (47), 8406-8419.
11. Crooks, G. E.; Hon, G.; Chandonia, J.-M.; Brenner, S. E., WebLogo: a sequence logo generator. *Genome Res.* **2004,** *14* (6), 1188-1190.
12. Thomen, P.; Lopez, P. J.; Bockelmann, U.; Guillerez, J.; Dreyfus, M.; Heslot, F., T7 RNA polymerase studied by force measurements varying cofactor concentration. *Biophys. J.* **2008,** *95* (5), 2423-2433.

13. Nicosia, A.; Tagliavia, M.; Costa, S., Regeneration of total RNA purification silica-based columns. *Biomed. Chromatogr.* **2010,** *24* (12), 1263-1264.
14. Aranda, P. S.; LaJoie, D. M.; Jorcyk, C. L., Bleach gel: a simple agarose gel for analyzing RNA quality. *Electrophoresis* **2012,** *33* (2), 366-369.
15. Team, R. C. *R: A language and environment for statistical computing.*, R Core Team: 2013.
16. Schindelin, J.; Arganda-Carreras, I.; Frise, E.; Kaynig, V.; Longair, M.; Pietzsch, T.; Preibisch, S.; Rueden, C.; Saalfeld, S.; Schmid, B.; Tinevez, J.-Y.; White, D. J.; Hartenstein, V.; Eliceiri, K.; Tomancak, P.; Cardona, A., Fiji: an open-source platform for biological-image analysis. *Nat. Methods* **2012,** *9* (7), 676-682.
17. Lawrence, A.-M.; Besir, H. U. S., Staining of proteins in gels with Coomassie G-250 without organic solvent and acetic acid. *J. Vis. Exp.* **2009,** *30* (30), 1350.
